# Guidelines for home-based enzyme replacement therapy in children and adolescents with mucopolysaccharidosis: a scoping review^*^

**DOI:** 10.1590/1980-220X-REEUSP-2025-0589en

**Published:** 2026-07-17

**Authors:** Vinicius Rodrigues de Oliveira, Bárbara Letícia de Queiroz Xavier, Carlos Vinícius de Souza Felipe, Isabele Gouveia Muniz de Alencar, Hallyson Leno Lucas da Silva, Francisca Marta de Lima Costa Souza, Jonas Sâmi Albuquerque de Oliveira

**Affiliations:** 1 Universidade Federal do Rio Grande do Norte, Programa de Pós-Graduação em Enfermagem, Natal, RN, Brazil.; 2 Universidade Federal do Rio Grande do Norte, Programa de Pós-Graduação em Saúde Coletiva, Natal, RN, Brazil.; 3 Universidade Federal do Rio Grande do Norte, Curso de Medicina, Natal, RN, Brazil.; 4 Universidade Federal do Rio Grande do Norte, Departamento de Enfermagem, Natal, RN, Brazil.

**Keywords:** Mucopolysaccharidoses, Enzyme Replacement Therapy, Child, Adolescent, Home Health Nursing

## Abstract

**Objective:**

To identify and map the main guidelines for carrying out home-based enzyme replacement therapy (ERT) administered to children and adolescents diagnosed with mucopolysaccharidosis.

**Method:**

A scoping review, conducted according to JBI Manual for Evidence Synthesis (Edition 2024). Twelve data sources were consulted using specific search strategies. Data were extracted using a specific form and analyzed using simple descriptive statistics and a qualitative PAGER approach.

**Results:**

The final sample consisted of 13 articles published between 2007 and 2020, mostly conducted in European countries, with a predominance of authors affiliated with the Royal Manchester Children’s Hospital. Five patterns were identified for consolidating qualified and safe care in the context of homebased ERT administered to children and adolescents with types I, II, IV-A, and VII mucopolysaccharidoses. The nurse emerges as an essential professional in all stages and activities presented.

**Conclusion:**

Despite the progress made regarding guidelines for the decentralization of ERT, challenges to its implementation still persist, among which the need for dissemination of technical and scientific knowledge stands out.

## INTRODUCTION

Mucopolysaccharidosis (MPS) is a group of genetic disorders resulting from the body’s inability to adequately produce the enzymes responsible for breaking down glycosaminoglycans (GAGs). These, in turn, perform essential biological functions and, when not broken down by lysosomal activity, accumulate in the body. This accumulation causes serious complications in several bodily systems, and can lead to death in the first few years of life. Currently, seven types of MPS are known, classified and cataloged according to the deficient enzyme^(1,2)^.

Treatment for MPS has evolved over time; however, there is still no definitive cure for the disease. Current therapeutic interventions aim to slow the progression of the disease, mitigate symptoms, and improve patients’ quality of life. Given this scenario, the choice of appropriate treatment should consider aspects such as the type of MPS, the patient’s age, neurological involvement, and associated clinical conditions. Furthermore, the treatment of choice should be initiated early, during childhood, a stage of the disease in which it is possible to prevent organic complications^(2,3)^.

One of these treatments is Enzyme Replacement Therapy (ERT), which, although not a definitive solution, is currently the most accessible. ERT emerged in the 1990s as a successful alternative for treating Gaucher disease, and was marketed in the 2000s for the purpose of treating types I, II, IV-A, VI, and VII MPS. In general terms, ERT aims to supply the deficiency of the enzyme missing in the body of the patient with MPS, through weekly or bi-weekly intravenous infusions. The administered enzyme binds to mannose-6-phosphate (M6P) receptors located on the cell surface, where it is directed to lysosomes, which in turn enable the degradation of GAGs^(4,5)^.

ERT administration is generally performed in hospitals or infusion centers and has an average duration of three to five hours. This scenario can create barriers to therapeutic adherence, cause school absences for the patient and lost work time for family members, besides significantly impacting family organization and, consequently, negatively affecting the quality of life of those involved^(6)^.

Considering these limitations, the possibility arises of conducting the ERT at home, a practice already implemented in North American and European countries. Home administration of ERT offers several benefits, including greater adherence to treatment, comfort and well-being, as well as promoting satisfaction with therapy and self-management of the disease^(7,8)^. Furthermore, home care is particularly good within the context of the pediatric service, since this environment presents itself as a space with the least trauma, more favorable to child well-being and development^(9)^.

Despite this evidence, in-home ERT is not yet a consolidated reality in Brazil. However, authors^(10)^ published a consensus of Brazilian experts that recognizes this practice as viable and safe in the context of types I, II and VI MPS. Similarly, the Brazilian Ministry of Health establishes, through Clinical Protocols and Therapeutic Guidelines (*PCDT*), guidelines related to ERT^(11,12)^. Nonetheless, these documents provide limited guidance regarding conducting ERT in a home setting.

Therefore, the objective of the study was to identify and map the main guidelines for carrying out home-based enzyme replacement therapy administered to children and adolescents diagnosed with mucopolysaccharidosis.

## METHOD

### Design of Study

This is a scoping review study, based on the methodological framework proposed by *JBI Manual for Evidence Synthesis* (*Edition* 2024)^(13)^ and reported in accordance with the guidelines of *Preferred Reporting Items for Systematic Reviews and Meta-Analyses for Scoping Reviews* (PRISMA-ScR)^(14)^. The protocol for this study was previously registered in *Open Science Framework* (OSF) and is available for consultation through the registry https://osf.io/qusb6/.

The following research question was adopted: what are the main guidelines for administering home-based enzyme replacement therapy to children and adolescents diagnosed with MPS? It was developed using the Population, Concept, and Context (PCC) strategy, in which: Population (P) – children and adolescents diagnosed with MPS; Concept (C) – guidelines for enzyme replacement therapy; Context (C) – home environment.

### Eligibility Criteria

The following were considered eligible as evidence: primary studies, secondary studies, official documents (guides, manuals, protocols), dissertations, theses, books, and proceedings of scientific conferences published in any year or language, provided they answer the research question. Evidence that did not have a full text available in its entirety was excluded, as were reflection studies, since they did not conform to the methodological aspects established for this review.

### Data Sources and Search Strategy

The research was conducted using 12 data sources with international scope. The conventional literature was collected from the following sources: Cumulative Index to Nursing and Allied Health Literature (CINAHL via EBSCO) Cochrane Library, Embase*,* Latin American and Caribbean Literature in Health Sciences (LILACS) Medical Literature Analysis and Retrieval System Online (Medline via Pubmed), Science Direct, Scopus, and Web of Science.

Regarding the search in the grey literature, it was carried out using the Catalog of Theses and Dissertations of the Coordination for the Improvement of Higher Education Personnel (CAPES) Foundation, and the search engine Google Scholar, of the National Institute for Health and Care Excellence (NICE) and of World Health Organization Institutional Repository for Information Sharing (WHO IRIS).

For formulating a highly sensitive search strategy according to each data source, the following steps were followed: I) Consultation of the Health Sciences Descriptors (DeCS), Medical Subject Headings (MeSH) and Emtree*;* II) Verification of Alternative terms/uncontrolled descriptors; III) Organization of descriptors and alternative terms in a table in the software Microsoft Office Word, 2016 version; IV) Preparation of a search strategy by associating terms with Boolean operators “AND”, “OR” or “NOT”. The final strategy according to each data source is presented in Chart 1.

**Chart 1:** Search strategies employed in the data sources used – Natal, RN, Brazil, 2025.

Conventional literature	
Data source	Search strategy
CINAHL	(child OR children OR adolescent OR teen OR teenager OR youth) AND (mucopolysaccharidoses OR mucopolysaccharidosis) AND (“Enzyme Replacement Therapies” OR “Replacement Therapies, Enzyme” OR “Replacement Therapy, Enzyme” OR “Therapies, Enzyme Replacement” OR “Therapy, Enzyme Replacement”) AND (“Therapy, Home Infusion” OR “Home Infusion Therapies” OR “Infusion Therapies, Home” OR “Therapies, Home Infusion” OR “Infusion Therapy, Home”) NOT (Neoplasm NOT “Glycogen Storage Disease Type II” NOT “Gaucher Disease” NOT “Fabry Disease”)
Cochrane Library	Child OR Children in Title Abstract Keyword OR Adolescence OR Adolescents OR ‘Adolescents, Female’ OR ‘Adolescent, Female’ OR ‘Adolescent, Male’ OR ‘Male Adolescents’ OR Youth OR Youths OR Teen OR Teens OR Teenager OR Teenagers in Title Abstract Keyword AND mucopolysaccharidoses OR mucopolysaccharidosis in Title Abstract Keyword AND ‘Enzyme Replacement Therapy’ OR ‘Enzyme Replacement Therapies’ OR ‘Replacement Therapies, Enzyme’ OR ‘Replacement Therapy, Enzyme’ OR ‘Therapies, Enzyme Replacement’ OR ‘Therapy, Enzyme Replacement’ in Title Abstract Keyword OR ‘Home Infusion Therapy’ OR ‘Therapy, Home Infusion’ OR ‘Home Infusion Therapies’ OR ‘Infusion Therapies, Home’ OR ‘Therapies, Home Infusion’ OR ‘Infusion Therapy, Home’ in Title Abstract Keyword NOT Neoplasm NOT ‘Glycogen Storage Disease Type II’ NOT ‘Gaucher Disease’ NOT ‘Fabry Disease’ in Title Abstract Keyword
Embase	(‘child’/exp OR child OR ‘children’/exp OR children OR ‘adolescent’/exp OR ‘adolescent’) AND ‘mucopolysaccharidosis’ AND (‘enzyme replacement’ OR ‘enzyme replacement therapy’) AND ‘home infusion therapy’ NOT ‘neoplasm’ NOT ‘glycogen storage disease type 2’ NOT ‘gaucher disease’ NOT ‘fabry disease’
LILACS	mh:((criança OR adolescente) AND (mucopolissacaridoses) AND (“Terapia de Reposição de Enzimas”) OR (“Terapia por infusões no domicílio”) AND NOT (neoplasia) AND NOT (“Doença de Depósito de Glicogênio Tipo II”) AND NOT (“Doença de gaucher”) AND NOT (“Doença de fabry”))
Medline	(“child”[MeSH Terms] OR “child”[All Fields] OR “children”[All Fields] OR “child s”[All Fields] OR “children s”[All Fields] OR “childrens”[All Fields] OR “childs”[All Fields] OR (“child”[MeSH Terms] OR “child”[All Fields] OR “children”[All Fields] OR “child s”[All Fields] OR “children s”[All Fields] OR “childrens”[All Fields] OR “childs”[All Fields]) OR (“adolescences”[All Fields] OR “adolescency”[All Fields] OR “adolescent”[MeSH Terms] OR “adolescent”[All Fields] OR “adolescence”[All Fields] OR “adolescents”[All Fields] OR “adolescent s”[All Fields]) OR “adolescent*”[All Fields] OR “teen*”[All Fields] OR “teenager*”[All Fields] OR “youth*”[All Fields] OR “adolescent* female”[All Fields] OR “adolescent* male”[All Fields]) AND (“mucopolysaccharidose”[All Fields] OR “mucopolysaccharidoses”[MeSH Terms] OR “mucopolysaccharidoses”[All Fields] OR “mucopolysaccharidosis”[All Fields])) OR (“mucopolysaccharidose”[All Fields] OR “mucopolysaccharidoses”[MeSH Terms] OR “mucopolysaccharidoses”[All Fields] OR “mucopolysaccharidosis”[All Fields])) AND “Enzyme Replacement Therapy”[All Fields]) OR “Enzyme Replacement Therapies”[All Fields] OR “replacement therapies enzyme”[All Fields] OR “replacement therapy enzyme”[All Fields] OR “therapies enzyme replacement”[All Fields] OR “therapy enzyme replacement”[All Fields]) AND “Home Infusion Therapy”[All Fields]) OR (“Home Infusion Therapy”[MeSH Terms] OR (“home”[All Fields] AND “infusion”[All Fields] AND “therapy”[All Fields]) OR “Home Infusion Therapy”[All Fields] OR (“therapy”[All Fields] AND “home”[All Fields] AND “infusion”[All Fields])) OR “Home Infusion Therapies”[All Fields] OR (“Home Infusion Therapy”[MeSH Terms] OR (“home”[All Fields] AND “infusion”[All Fields] AND “therapy”[All Fields]) OR “Home Infusion Therapy”[All Fields] OR (“infusion”[All Fields] AND “therapies”[All Fields] AND “home”[All Fields])) OR (“Home Infusion Therapy”[MeSH Terms] OR (“home”[All Fields] AND “infusion”[All Fields] AND “therapy”[All Fields]) OR “Home Infusion Therapy”[All Fields] OR (“therapies”[All Fields] AND “home”[All Fields] AND “infusion”[All Fields])) OR “infusion therapy home”[All Fields]) NOT (“neoplasm”[All Fields]) NOT “Glycogen Storage Disease Type II”[All Fields]) NOT “Gaucher Disease”[All Fields]) NOT “Fabry Disease”[All Fields]
Science Direct	(Child AND Mucopolysaccharidoses AND “Enzyme Replacement Therapy” AND “Home Infusion Therapy” NOT Neoplasm NOT “Glycogen Storage Disease Type II” NOT “Gaucher Disease” NOT “Fabry Disease”)
Scopus	(TITLE-ABS-KEY (child OR children OR adolescent OR teen OR teenager OR youth ) AND TITLE-ABS-KEY (mucopolysaccharidoses OR mucopolysaccharidosis ) AND TITLE-ABS-KEY (“Enzyme Replacement Therapies” OR “Replacement Therapies, Enzyme” OR “Replacement Therapy, Enzyme” OR “Therapies, Enzyme Replacement” OR “Therapy, Enzyme Replacement” ) AND TITLE-ABS-KEY (“Therapy, Home Infusion” OR “Home Infusion Therapies” OR “Infusion Therapies, Home” OR “Therapies, Home Infusion” OR “Infusion Therapy, Home” ) AND NOT TITLE-ABS-KEY (neoplasm AND NOT “glycogen storage disease type iii” AND NOT “Gaucher Disease” AND NOT “Fabry Disease”))
Web of Science	child OR children OR adolescent OR Teen OR Teenager OR Youth (All Fields) AND Mucopolysaccharidoses OR Mucopolysaccharidosis (All Fields) AND ‘Enzyme Replacement Therapies’ OR ‘Replacement Therapies, Enzyme’ OR ‘Replacement Therapy, Enzyme’ OR ‘Therapies, Enzyme Replacement’ OR ‘Therapy, Enzyme Replacement’ (All Fields) AND ‘Therapy, Home Infusion’ OR ‘Home Infusion Therapies’ OR ‘Infusion Therapies, Home’ OR ‘Therapies, Home Infusion’ OR ‘Infusion Therapy, Home’ (All Fields) NOT Neoplasms NOT ‘Glycogen Storage Disease Type II’ NOT ‘Gaucher Disease’ NOT ‘Fabry Disease’ (All Fields)
Gray literature	
Data source	Search strategy
CAPES Catalog of Theses & dissertations	(Criança OR Adolescente) AND Mucopolissacaridoses AND (Terapia de Reposição de Enzimas OR Terapia de Reposição Enzimática) AND Terapia por Infusões no Domicílio.
Google Scholar	Child OR Adolescent AND Mucopolysaccharidoses AND “Enzyme Replacement Therapy” AND “Home Infusion Therapy”
NICE	Child OR Adolescent AND Mucopolysaccharidoses AND “Enzyme Replacement Therapy” AND “Home Infusion Therapy”
WHO IRIS	Child OR Adolescent AND Mucopolysaccharidoses AND “Enzyme Replacement Therapy” AND “Home Infusion Therapy”

Source: Prepared by the authors.

Despite the operator “NOT” not being conventionally used, due to the risk of limiting the review sample, its inclusion proved necessary, since ERT can be developed in other disease contexts. Thus, during testing for the construction of the search strategy, it was noticed that, without the use of this operator, numerous studies emerged that did not address ERT in children and adolescents with MPS, generating an overload of results that did not fit the scope of the research.

The data search took place on February 21, 2025, through the CAPES Periodicals Portal, accessing the Federated Academic Community (CAFe), and was conducted in pairs by two researchers. It is important to highlight that, before starting the data search and collection phase, all research members underwent prior training, given by the principal investigator, with the aim of ensuring the standardization of procedures and obtaining more reliable results from the team.

### Data Extraction

The publications found in the data sources were exported to the Rayyan software^(15)^ (version 2024, Computing Research Institute, Doha, Qatar), where the initial screening was held considering the previously established eligibility criteria, as well as the reading of titles and abstracts. The selection was made by the same two researchers, who participated in the previous stage and so that there would be no influence on the material selection, it was conducted blindly, meaning the reviewers did not have access to the other opinions until this stage was finalized.

Regarding conflicts whether to include or not a particular study in the sample, the aim was initially to find a solution through discussion among the evaluators. In cases where disagreement persisted, the decision was made based on the issuance of the final opinion of a third evaluator linked to the research team, this being Nurse with a PhD in Nursing and experience in caring for children and adolescents with MPS.

After assessing the eligibility of the studies found in the data sources and resolving any conflicts, a manual search for references was undertaken. This step consisted of analyzing the bibliographic lists of the articles selected as part of the final sample, to identify additional references that could contribute to a deeper understanding of the study.

Data extraction from the studies selected for the sample was performed independently, using the platform *Google Forms*, using a customized form, specifically designed to meet the objectives of this review.

The form was organized into two sections. The first section involved extracting data related to the bibliometric profile of the studies in the sample, in which information was collected regarding the authors, affiliated institution, journal, year of publication of the studies, country where the research was conducted, objective, type of document, and type of study. The second section addressed the content related to guidelines for home-based ERT administered to children and adolescents with MPS.

### Data Analysis and Treatment

The data obtained were analyzed quantitatively using simple descriptive statistics (relative and absolute frequencies) to describe the bibliometric profile of the studies included in the sample, and qualitatively using the PAGER framework. This strategy involves a systematic approach developed to improve the analysis and reporting of scoping reviews.

The PAGER structure is a mnemonic that represents five interrelated analytical domains: Standards, Advances, Gaps, Evidence for Practice, and Research Recommendations. This approach allows for a clear and accessible mapping of key thematic findings, identification of progress in the field of study, highlighting shortcomings in the existing body of literature, and emphasizing practical implications and future research directions^(16)^.

## RESULTS

### Presentation of The Selection of Studies

**Figure 1:**
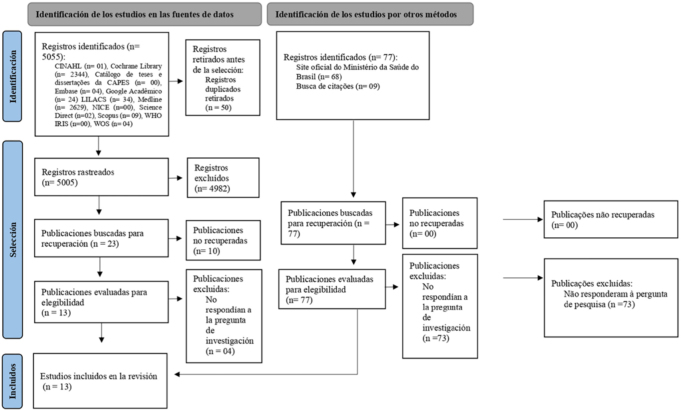
Flowchart for selecting studies according to PRISMA recommendations (2020). Natal, RN, Brazil, 2025.

A total of 5132 records were identified, of which 5055 originated from search strategies in data sources, resulting in nine included studies. A further 77 records were identified using additional methods, of which four were selected. The final sample consisted of 13 articles, and the detailed selection of studies is presented in Figure 1.

### Bibliometric Profile and Detailed Description of The Studies that Comprised The Sample

The reviewed articles were published between 2007 and 2020, but with gaps in the years 2012, 2014, 2016, and 2019. A higher concentration of publications was observed in the years 2008, 2010, and 2018, each representing 15.4% of the sample (n = 2), totaling 46.2% of the included studies. In the other years in which there were publications, the frequency was approximately 7.7% (n = 1).

Regarding geographical distribution, it is observed that most studies originate from European countries, which together represent approximately 69.3% (n = 9) of the sample, with the United Kingdom and Italy standing out. Subsequently, the participation of countries from the American continent, such as Brazil, the USA, and Canada, stands out.

Regarding the databases used for retrieving the studies, *Scopus*, *Medline* and *Google* Scholar studies stood out, comprising 69% (n = 9) of the sample; the remaining studies were published on the Science Direct platform, on the official website of the Ministry of Health, and in the Scientific Electronic Library Online (SciELO). It should be noted that, although SciELO was not used as a data source in this review, one of the articles included in the sample, identified through a manual search of the references, was available in that database.

With regard to the journals in which the studies were published, it became evident that the journal Molecular Genetics and Metabolism gathered the largest number of publications, corresponding to approximately 23.1% (n = 3) of the sample. This journal is classified as high-impact, according to metrics for the year 2024; on Scopus database*,* it has a percentile of 65% and an impact factor of 3.5, according to the analysis of the Journal Citation Reports (JCR). The remaining journals had an individual participation of 7.7% (n = 1).

Regarding the authors’ institutional affiliations, it was possible to group the institutions by core categories, namely: hospitals and other health institutions, universities and health agencies, or specialist councils/societies. Thus, it was identified that the authors who published the most in the area of home ERT were linked to hospitals (50%), especially the Royal Manchester Children’s Hospital with a greater number of professionals linked to the articles in the sample.

The description of the content of the studies selected for the final sample of the review is presented in Chart 2.

**Chart 2:** Description of the studies selected for the sample - Natal, RN, Brazil, 2025.

Author	Design of study	Sample size	Objective	Results
Wikman-Jorgensen et al^(17)^	Systematic review with meta-ana lysis	42 studies	To evaluate the effectiveness of ERT in Hunter syndrome in terms of its impact on clinical and safety variables.	Data suggest a clear and consistent effect of ERT on Hunter syndrome.
Brasil^(11)^	Clinical Protocol and Therapeutic Guideline	N/l*	To review diagnostic and therapeutic practices and incorporate recommendations regarding drug treatment with laronidase-based ERT.	It presents the general concept of type I MPS and establishes diagnostic criteria, inclusion and exclusion criteria, treatment, and mechanisms for regulation, control, and evaluation.
Brasil^(12)^	Clinical Protocol and Therapeutic Guideline	N/l*	To review diagnostic and therapeutic practices and incorporate recommendations regarding drug treatment with idursulfase alpha ERT.	It presents the general concept of Type II MPS and establishes diagnostic criteria, inclusion and exclusion criteria, treatment, and mechanisms for regulation, control, and evaluation.
Finnigan et al.^(18)^	Experience Report	14 participants	To review the experience of a UK pediatric setting with home-based ERT treatment in children with MPS type IV-A and to demonstrate the criteria required to ensure a successful home treatment program.	Two patients had to return to the hospital due to difficulties with venous access at home; one returned home after being fitted with a fully implanted catheter, and the other resumed infusions at home with venous access. Another patient developed an adverse infusion-related reaction, so he returned to the hospital setting for two infusions while his premedication regimen and rate increments were altered. This patient did not experience any further reactions within the home care setting.
Sestito et al.^(19)^	Literature review	N/l*	To conduct a literature review on idursulfase treatment in patients with Hunter syndrome.	The efficiency and security of idursulfase therapy have been evaluated and confirmed in many clinical reports. Long-term follow-up of patients on ERT has demonstrated the importanceof theearly beginning of idursulfase treatment. Regarding home-based therapy, it has been associated with increased patient adherence compared to hospital treatment and an improvement in the quality of life for both patients and their families.
Ceravo Io et al.^(20)^	Experience Report	3 participants	To report the results of a trial in southern Italy with 3 patients with Hunter syndrome who have been receiving home treatment for 73 to 87 weeks.	During the first year of home treatment, more infusions were administered at home than at the specialized center. Parental scores on the Health and Wellbeing Questionnaire (EQ-5D) increased when the patient was receiving home treatment, indicating a significant improvement in quality of life.
Scarpa et al.^(21)^	Expert consensus and literature review	26 specialists	To describe the recommendations developed by the European Hunter Syndrome Expert Council for the diagnosis and treatment of MPS type II.	The recommendations emphasize the need for a multidisciplinary approach. ERT with idursulfase helps improve somatic signs, but has no effect on neurological symptoms. Home-based therapy promotes greater patient adherence, as well as a better quality of life. However, the transition to home should be evaluated according to strict protocols.
Burton et al.^(22)^	Experience Report	11 participants	To report the experience with 11 patients with MPS disorders on ERT who were successfully transferred to home infusions for periods ranging from 21 to 86 weeks.	Home-based ERT is safe for patients with MPS. A statistically significant difference was observed in the number of missed infusions in the infusion center versus the home setting. All patients expressed satisfaction with the at-home therapy, and none of them experienced any adverse reactions during home ERT. Eight of the patients who attend school have returned to a full-time school schedule.
Giugliani et al.^(10)^	Expert consensus	47 specialists	To gather and harmonize available information on the treatment of MPSs types I, II and IV.	It provides guidance on ERT for the treatment of MPS in Brazil.
Burton et al.^(23)^	Experience Report	92 participants	To use patient experience from the HOS - Hunter Outcome Survey observational database to assess the feasibility of home infusions of idursulfase for patients with MPS type II.	Patients initiated home ERT after a median of 9 months on idursulfase. Most were between 5 and 11 years old at the time of the transfer. Six patients discontinued home therapy; four subsequently resumed home infusions. Five infusion-related reactions occurred in 2 of the 59 patients who received home therapy for at least 12 months. The reactions were classified as mild to moderate. All reactions that occurred at home were promptly treated at home.
Bagewadi et al.^(24)^	Experience Report	23 participants	To review the authors' experience with home-based ERT treatment in MPSs types II and IV.	Both Elaprase and Naglazyme can be safely administered in the patient's home, thus easing the burden of treatment. When deciding on the suitability of home treatment, a robust protocol with careful patient selection, good vascular access, and a detailed treatment plan for adverse reactions and anaphylaxis are essential for successful home treatment.
Cox-Brinkman et al.^(25)^	Cohort study	17 participants	To investigate the feasibility and safety of home-based therapy for patients with MPS type I in the Netherlands.	ERT can be administered satisfactorily and safely at home in both children and adults with MPS type I. Over 1000 home infusions have resulted in no infusion-related reactions or prolonged technical difficulties. Only two patients experienced an infusion-related reaction during the initial hospital ERT period. None of the patients experienced any infusion-related reaction after 3 months of ERT.
Wraith et al.^(26)^	Clinical review	N/l*	To provide an overview of the clinical manifestations, diagnosis, and symptomatic treatment of patients with MPS type II and to provide recommendations for the use of ERT.	ERT with idursulfase has been shown to significantly reduce urinary GAG levels and improve clinical symptoms of MPS, especially when initiated early. Although ERT does not cross the blood-brain barrier, it represents an important advance in the management of somatic disease. Treatment should be multidisciplinary, initiated as early as possible, and tailored to the patient's clinical needs, including home-based options for stable cases.

Legend: ^*^Not identified in the study.

Source: Prepared by the author.

### Main Guidelines for Carrying out Home-Based Enzyme Replacement Therapy Administered to Children and Adolescents Diagnosed with Mucopolysaccharidosis

Analysis of the sample allowed us to identify five fundamental patterns for the development of qualified and safe assistance in home-based ERT administered to children and adolescents with MPS. Based on each of the established standards, progress, gaps, evidence, and, above all, the main guidelines for administering home-based ERT were observed. Chart 3 summarizes the main findings organized according to the PAGER strategy.

**Chart 3:** PACER Structure applied to the context of the study - Natal, RN, Brazil, 2025.

Standards	Advances	Gaps	Evidence for practice	Recommendations
Prior assessment of the child or adolescent to make the decision to initiate home-based ERT, based on indications and contraindications(^10,12,17,18,19,20,21,22,23,24,25,26)^).	Development of protocols to assist in decision-making regarding the transfer of a child or adolescent to a home-based ERT.	Lack of standardization in the literature regarding the minimum age and prior period of time performing ERT in a hospital/outpatient setting before initiating it at home.	Home-based ERT can be initiated if the child or adolescent meets all of the following criteria: - Age five years or older (preferably). - Started ERT at least six months ago in an outpatient/hospital setting, without experiencing any adverse infusion-related reactions. - Presence of peripheral venous access that is easy to puncture or alreadyt has a fully implanted catheter. - Has a home environment that is favorable for conducting the ERT. - Does not have significant respiratory disease.	Conducting studies with a higher level of scientific evidence that allow us to define the ideal age to transition a child to homebased ERT.
Guidelines for the family and the patient before starting home-based ERT.(^18,20,22,23,24^).	Guidance provided by professionals from different fields and specialties.	Most studies provide little detail regarding the guidance that should be given to the family of a child or adolescent who will begin home-based ERT.	Family members should be given guidance regarding: - The possibility of starting administering infusions at home only when the parents feel comfortable and agree to the treatment being carried out at home. - The need for a suitable environment to carry out all the processes involved in infusion. - Benefits of home-based ERT. - Risks of infusion-related reactions and measures for managing these reactions. - Possible risks associated with a fully implanted catheter. - Factors that may lead to interruption of home treatment.	To broaden the socio-familial perspective in studies dealing with home-based ERT, presenting a more comprehensive approach to the role of the caregiver and the importance of guidance for family members.
Preparation and administration of medication for home-based ERT.(^10,11,12,18,24^).	No progress was identified regarding the preparation and administration of ERT at home. The process generally follows the same protocols established for performing ERT in an outpatient setting.	There is no comprehensive description of the aspects required for a home environment to be considered ideal for preparing for and administering ERT. The studies do not mention personalized preparation and administration of medication for the home environment. The pattern is followed, depending on whether it is an outpatient or inpatient setting.	- Approval, by the medical and nursing team, of the location for medication preparation and storage. - Before starting the infusion, the professional who will be monitoring the procedure must: a) Conduct and record medical history and physical examination. b) Administer premedication to prevent adverse infusion reactions, as prescribed by a physician. - Proper aseptic techniques must be used during the preparation and administration process. - Regarding preparation, one should: a) Select the number of medication vials considering the patient's weight and the recommended standard dose. b) Place the vials outside of refrigeration until they reach room temperature naturally, without heating them. c) Check the appearance of the medication. If the result is altered, the infusion should not be performed. d) Determine the total volume the patient will receive during the infusion. e) Discard an amount of saline solution from the bag equivalent to the volume that will be taken from the enzyme vials, ensuring that, after the addition of the enzyme, the total volume of the solution remains 100 mL or 250 mL. f) Slowly remove the appropriate amount of enzyme from the vials. It is important that the solution is not agitated, as agitation can denature the product and compromise its biological activity. g) Gradually add the enzyme to the bag containing saline solution. The bag containing the prepared solution should be gently shaken to ensure that the medication is distributed evenly. - The medication should be administered immediately after preparation. If this is not possible, the solution should not be left at room temperature. It is recommended to store it in a refrigerator, between 2°C and 8°C. - The infusion should be administered intravenously using an infusion pump. Appropriate equipment for the device must be used, along with a 0.2 μm filter.	Development and validation of manuals or protocols to guide the administration of ERT, considering the differences between home and hospital settings.
Nursing care for home-based ERT(^10,18,20,21 ,22,23,24,25^).	Training to improve the quality of service.	- Lack of detail regarding the content of the nurses' training. - The number of patients a home care nurse can manage is not specified.	Nurses are responsible for: - Observing an infusion session while the patient is still being treated in the hospital. - Conduct a preliminary visit to the patient's home and verify the suitability of the environment for performing the ERT and storing the medications. - To hold a meeting with family members, provide them with guidance, and answer their questions. Prepare and administer ERT medication. - Monitor the entire infusion session at home. Manage adverse reactions. Submit a monthly clinical report to monitor the clinical progression of the patients' disease. - Always stay in contact with the infusion center, especially in cases of adverse reactions. - Report the progress of the infusions and any adverse reactions to the primary healthcare physician.	To establish clearer regulations regarding the training and work-related aspects of the role of nurses providing care to children and adolescents in homebased ERT.
Management of adverse reactions(^10,12,18,19,22,23,24,26^).	Adverse reaction management network	There is no specification of time intervals in which each measure should be taken, or at what point the patient should be reassessed for the implementation of the next action.	-The nurse must be adequately equipped to manage possible adverse reactions. - In cases of mild reactions, such as chills, itching, nausea, headache and/or irritability (especially in younger children), the professional responsible for the infusion should: a) Decrease the infusion rate by 50%. b) Administer an antipyretic and antihistamine orally. c) If the patient still presents symptoms, reduce the infusion rate by a further 50%. d) If symptoms persist, discontinue the infusion permanently. - In cases of moderate reactions, such as fever, severe headache, vomiting, chest pain, abdominal pain and/or hives, the professional responsible for the infusion should: a) Stop the infusion immediately. b) Administer antihistamines and corticosteroids intravenously. c) Administer a bronchodilator, if previously prescribed by a doctor. - In cases of severe reactions, such as dyspnea, tongue or glottis edema, anaphylactic or cardiogenic shock, the professional monitoring the infusion should: a) Stop the infusion immediately. b) Call the emergency services. c) Administer epinephrine intramuscularly, in addition to antihistamines and corticosteroids intravenously. d) Stabilize and transfer to the hospital. e) Collect blood for IgE testing. - In cases of moderate or severe adverse reactions, the next infusion should take place in an outpatien/hospital setting.	Creation or adaptation of clinical monitoring instruments for application in the context of adverse reactions related to ERT.

Source: Prepared by the author.

A particularly relevant finding in this review is the identification of guidelines for home therapy specifically for patients diagnosed with MPS type I^(10,11,17,25)^; MPS type II^(10,12,19,20,21,22,24,26)^; MPS type IV-A^(18,22,24)^; MPS type VI^(10,22)^. Therefore, the guidelines presented here are limited to these types of MPS.

## DISCUSSION

The results of this study allowed for the identification and mapping of the main guidelines that underpin the decentralization of ERT, administered to children and adolescents, to the home environment. This evidence contributes to the safe implementation of home-based ERT, by highlighting aspects that can support the practice and decision-making of healthcare professionals involved in this process, with an emphasis on the role of the nurse.

The transition to home care requires a thorough assessment of multiple aspects, among which the patient’s clinical condition and the home environment stand out as paramount^(27)^. In the context of ERT, the aforementioned factors remain essential; however, other mandatory elements are added that take into account the specific characteristics of the child or adolescent with MPS^(18)^. In the case of pediatric patients, the first step is to identify their age range to determine if a transition to home care is recommended.

Based on the experience of authors^(22)^, a safe transition of ERT to home therapy for patients under five years old is possible, while other authors^(19,21,24)^ acknowledge that there is a lack of solid evidence to support the decision to initiate homebased ERT in children under five years of age; however, these authors do not rule out the possibility of treatment occurring at home, provided there is a thorough medical evaluation to support the decision.

The number of infusions previously performed in a hospital setting should also be taken into consideration. It is recommended that the child or adolescent has gone through at least three^(19,21,22,24,26)^ to six months^(11,12,22,25)^ of treatment in a hospital setting, since adverse reactions are common during the first few months of administration.

Although no studies have been found that portray the Brazilian experience in the context of home-based ERT, professionals can follow the recommendations of the PCDTs^(11,12)^ for MPS, of the Ministry of Health, which recommend that at least six months of therapy be completed in a hospital setting before treatment can be transferred to the home.

Another factor that may influence the decision to administer ERT at home is whether the patient has a fully implanted catheter. Authors^(25)^ report that, despite interest in providing home-based therapy, the difficulty of obtaining peripheral venous access led to the exclusion of adolescents from this treatment model.

The use of a catheter becomes necessary because obtaining peripheral access is difficult due to bodily changes resulting from MPS^(28)^. It is also considered that the use of a totally implanted catheter, in addition to facilitating patient management during home therapy, can increase adherence to treatment, since it provides less stressful experiences for the child.

To confirm the feasibility of decentralizing treatment to the home, it is still necessary to verify that there are no signs of risk or factors that could compromise the treatment. Studies advise against home-based ERT for patients with significant respiratory disease, as these conditions present a higher risk of complications and make their management more difficult. A classification of respiratory disease as significant can be obtained through spirometric evaluation when the forced vital capacity (FVC) test result is 40% or less, or when evidence of severe obstructive airway disease is observed^(19,21,24,26)^.

Once the profile verification stage is complete, whether the family is interested in participating in this therapeutic modality should be checked. Studies have shown that, when given the opportunity, most families opt for home treatment, being willing to collaborate in various aspects, including assisting the nurse during the infusion^(24,25)^. However, there are some cases in which family members of sick children and adolescents choose to continue treatment in a hospital setting, a decision often linked to a feeling of unpreparedness to deal with the healthcare demands^(29)^.

Therefore, the need for clear guidance for family members emerges as a central point in the care transition process. First and foremost, it is essential to ensure that the home has a suitable environment, which is why it is recommended that the doctor or nurse make a home visit to assess whether all the necessary requirements for the infusion are met, namely: a place to store the medication, space to perform the infusion, and issues related to the professional’s safety^(10,24)^. In Brazil, these recommendations are ratified by the Brazilian Health Regulatory Agency^(30)^.

Family members should also be properly informed about the benefits of home therapy, such as reduced expenses, less travel, reduced losses related to work and school activities, greater patient comfort, and improved quality of life^(17,20,22,23)^.

Another guideline refers to the risk of adverse reactions. Although these occur in a small number of patients, there is a possibility of manifestation, especially in younger children. The family should be informed that home treatment may be temporarily interrupted in the event of moderate or severe adverse reactions^(21,24)^.

After preparing the child, adolescent, and their family, the steps related to administering the ERT begin. Based on the studies analyzed in this review, no substantial differences were identified between the preparation and administration of ERT in the hospital and at home. The recommendations are to initially conduct a medical history and physical examination of the patient, followed by the administration of medication prior to the infusion. Subsequently, the enzymes are prepared using aseptic techniques, rigorous hand hygiene, and attention to the appearance of the medication^(10)^.

It is important to highlight that, although these practices are validated among experts, it is necessary to consider aspects related to the home environment, which can present various nuances. Therefore, it is recommended to develop tools that guide the proper practice of enzyme preparation and administration, considering this environment.

Based on the recommendations presented here, it can be stated that nurses play important roles in the ERT process. In international literature, the nurse is identified as an essential figure for the success of ERT and her close relationship with the patient contributes to a better experience during treatment^(18,20,22,24,25)^. In addition to the actions already presented, the nurse’s responsibilities also include managing potential adverse reactions, submitting monthly clinical reports, and maintaining regular contact with the primary care physician.

Thus, it is observed that the studies that comprised this review point to the understanding that home-based ERT demands the development of Advanced Nursing Practice. Advanced practice nurses are professionals who possess a framework of specialized knowledge and develop skills for complex clinical decision-making^(31)^.

Regarding the management of adverse reactions, the nurse will act according to the severity of the reaction, which can be mild, moderate, or severe. In mild cases, decreasing the infusion rate combined with the use of antipyretics and antihistamines is usually sufficient. For cases demonstrating greater severity, interruption of the infusion is recommended with the combination of medications from different classes^(24)^. In cases of severe reactions, the nurse is also responsible for collecting a blood sample for IgE testing, assessing the possibility of initiating drug tolerance protocols, conducted within the hospital setting^(11,12)^.

One highlight was the creation of the adverse reaction management network, an experience reported by the authors^(20)^. This network is composed of hospitals, primary care physicians, nurses, and patients’ family members, all integrated into the home-based ERT.

In addition to clinical aspects, the nurse is responsible for managing issues, such as producing a weekly report on the ERT, forwarding it to the infusion center, and maintaining continuous communication with the primary care physician. This coordination reaffirms the guidelines of the National Policy for Comprehensive Care for People with Rare Diseases^(32)^. Given this scenario, it is essential to point out that the multiple responsibilities of nurses and the demand for complex care can lead to exhausting routines. This reinforces the need to establish specific guidelines for the nurse’s role in the context of home-based ERT.

Regarding the study’s limitations, the authors acknowledge that the literature on home-based ERT administered to children and adolescents with MPS is scarce, a common characteristic in research dealing with therapies for rare diseases. The age range limitation may have further reduced the identification of studies, especially those that include populations of different ages. Furthermore, the potential restriction of access to articles not freely available, even when searching through the CAPES Periodicals Portal, may have limited the scope of the search, resulting in the exclusion of potentially relevant evidence.

Moreover, the absence of more recent publications was observed, that is, in the period from 2021 to 2024, which demonstrates a gap in the literature and reinforces the need for new research that produces updated scientific evidence on home-based ERT associated with the context of childhood and adolescence.

This study contributes to the advancement of nursing by highlighting the leading role of nurses in the administration of ERT, both in hospital settings and at home. It also provides subsidies to support professionals in the process of decentralizing ERT to home care, while raising debate about the challenges involved in the transition of children and adolescents to this new care context.

## CONCLUSION

Home-based ERT is already being implemented in several countries around the world and has seen growing positive experiences in Latin American countries, but is still timid in Brazil. Although home care has been established in the country over the years, its association with ERT requires a more comprehensive understanding of MPS and its clinical management, which is often limited to a small group of specialist professionals.

Although progress has been identified in the articulation of ERT in the home setting, ranging from defining parameters for patient eligibility to the management and execution of care at home, significant gaps still persist that need to be overcome at the national level, such as the lack of systematization of this knowledge for implementation in healthcare practice.

Finally, it is worth highlighting that the nurse was identified as a key figure in the home-based ERT process. Thus, given the complexity and scope of the responsibilities performed by this professional in this context, the need to strengthen Advanced Nursing Practice becomes evident, to support qualified clinical decision-making. Additionally, the need for the development of protocols and other care tools to guide the practice of homebased ERT is highlighted.

## Data Availability

All the data supporting the results of this study were published in the article itself.

## References

[1] Nagpal R, Goyal RB, Priyadarshini K, Kashyap S, Sharma M, Sinha R (2022). Mucopolysaccharidosis: a broad review.. Indian J Ophthalmol.

[2] Michaud M, Belmatoug N, Catros F, Ancellin S, Touati G, Levade T (2020). Mucopolysaccharidoses: quand y penser?. Rev Med Interne.

[3] Taylor M, Khan S, Stapleton M, Wang J, Chen J, Wynn R (2019). Hematopoietic stem cell transplantation for mucopolysaccharidoses: past, present, and future.. Biol Blood Marrow Transplant.

[4] Parini R, Deodato F (2020). Intravenous enzyme replacement therapy in mucopolysaccharidoses: clinical effectiveness and limitations.. Int J Mol Sci.

[5] Chen HH, Sawamoto K, Mason RW, Kobayashi H, Yamaguchi S, Suzuki Y (2019). Enzyme replacement therapy for mucopolysaccharidoses: past, present, and future.. J Hum Genet.

[6] Penon-Portmann M, Blair DR, Harmatz P (2023). Current and new therapies for mucopolysaccharidoses.. Pediatr Neonatol.

[7] Heinrich R, Claus F, Schoenfelder T (2023). The patients’ perspective on home-based infusion: a longitudinal observational study in the German healthcare setting for patients with lysosomal storage disorders treated with enzyme replacement therapy.. Mol Genet Metab Rep.

[8] Gorski LA (2019). The impact of home infusion therapies on caregivers.. Semin Oncol Nurs.

[9] Castor C, Landgren K, Hansson H, Kristensson Hallström I (2018). A possibility for strengthening family life and health: family members’ lived experience when a sick child receives home care in Sweden.. Health Soc Care Community.

[10] Giugliani R, Federhen A, Rojas MVM, Vieira TA, Artigalás O, Pinto LLC (2010). Terapia de reposição enzimática para as mucopolissacaridoses I, II e VI: recomendações de um grupo de especialistas brasileiros.. Rev Assoc Med Bras.

[11] Brasil. (2018). Ministério da Saúde. Portaria Conjunta nº 12, de 11 de abril de 2018. Aprova o Protocolo Clínico e Diretrizes Terapêuticas da Mucopolissacaridose do tipo I..

[12] Brasil. (2018). Ministério da Saúde. Portaria Conjunta nº 16, de 24 de maio de 2018. Aprova o Protocolo Clínico e Diretrizes Terapêuticas da Mucopolissacaridose do tipo II..

[13] Peters MDJ, Godfrey C, McInerney P, Munn Z, Tricco AC, Khalil H, Aromataris E, Lockwood C, Porritt K, Pilla B, Jordan Z (2024). JBI manual for evidence synthesis..

[14] Tricco AC, Lillie E, Zarin W, O’Brien KK, Colquhoun H, Levac D (2018). PRISMA Extension for Scoping Reviews (PRISMA-ScR): checklist and explanation.. Ann Intern Med.

[15] Ouzzani M, Hammady H, Fedorowicz Z, Elmagarmid A (2016). Rayyan: a web and mobile app for systematic reviews.. Syst Rev.

[16] Bradbury-Jones C, Aveyard H, Herber OR, Isham L, Taylor J, O’Malley L (2021). Scoping reviews: the PAGER framework for improving the quality of reporting.. Int J Soc Res Methodol.

[17] Wikman-Jorgensen PE, López Amorós A, Peris García J, Esteve Atienzar PJ, Cañizares Navarro R, Asensio Tomás ML (2020). Enzyme replacement therapy for the treatment of Hunter disease: a systematic review with narrative synthesis and meta-analysis.. Mol Genet Metab.

[18] Finnigan N, Roberts J, Mercer J, Jones SA (2017). Home infusion with elosulfase alpha (Vimizim®) in a UK paediatric setting.. Mol Genet Metab Rep.

[19] Sestito S, Ceravolo F, Grisolia M, Pascale E, Pensabene L, Concolino D (2015). Profile of idursulfase for the treatment of Hunter syndrome.. Res Rep Endocr Disord.

[20] Ceravolo F, Mascaro I, Sestito S, Pascale E, Lauricella A, Dizione E (2013). Home treatment in paediatric patients with Hunter syndrome: the first Italian experience.. Ital J Pediatr.

[21] Scarpa M, Almássy Z, Beck M, Bodamer O, Bruce IA, de Meirleir L (2011). Mucopolysaccharidosis type II: european recommendations for the diagnosis and multidisciplinary management of a rare disease.. Orphanet J Rare Dis.

[22] Burton BK, Guffon N, Roberts J, van der Ploeg AT, Jones SA (2010). Home treatment with intravenous enzyme replacement therapy with idursulfase for mucopolysaccharidosis type II – data from the Hunter Outcome Survey.. Mol Genet Metab.

[23] Burton BK, Wiesman C, Paras A, Kim K, Katz R (2009). Home infusion therapy is safe and enhances compliance in patients with mucopolysaccharidoses.. Mol Genet Metab.

[24] Bagewadi S, Roberts J, Mercer J, Jones S, Stephenson J, Wraith JE (2008). Home treatment with Elaprase and Naglazyme is safe in patients with mucopolysaccharidoses types II and VI, respectively.. J Inherit Metab Dis.

[25] Cox-Brinkman J, Timmermans RG, Wijburg FA, Donker WE, van de Ploeg AT, Aerts JM (2007). Home treatment with enzyme replacement therapy for mucopolysaccharidosis type I is feasible and safe.. J Inherit Metab Dis.

[26] Wraith JE, Scarpa M, Beck M, Bodamer OA, de Meirleir L, Guffon N (2008). Mucopolysaccharidosis type II (Hunter syndrome): a clinical review and recommendations for treatment in the era of enzyme replacement therapy.. Eur J Pediatr.

[27] Brasil. (2020). Ministério da Saúde. Atenção Domiciliar na Atenção Primária à Saúde..

[28] Yildiz Y, Sivri HS (2021). Difficulties associated with enzyme replacement therapy for mucopolysaccharidoses.. Turk Arch Pediatr.

[29] Silva-Rodrigues FM, Bernardo CSG, Alvarenga WA, Janzen DC, Nascimento LC (2019). Transição de cuidados para o domicílio na perspectiva de pais de filhos com leucemia.. Rev Gaúcha Enferm.

[30] Brasil (2006). Agência Nacional de Vigilância Sanitária – ANVISA. Resolução RDC nº 11, de 26 de janeiro de 2006. Dispõe sobre o regulamento técnico de funcionamento de serviços que prestam atenção domiciliar..

[31] International Council of Nurses – ICN. (2020). Guidelines on advanced practice nursing.. https://www.icn.ch/resources/publications-and-reports/guidelines-advanced-practice-nursing-2020.

[32] Brasil (2014). Ministério da Saúde. Portaria nº 199, de 30 de janeiro de 2014. Institui a Política Nacional de Atenção Integral às Pessoas com Doenças Raras..

