# Supplementary material for: Guidelines for home-based enzyme replacement therapy in children and adolescents with mucopolysaccharidosis: a scoping review
Source: Rev Esc Enferm USP. 2026 Jul 17;60:e20250589. doi: 10.1590/1980-220X-REEUSP-2025-0589en (PMC13398687; doi:10.1590/1980-220X-REEUSP-2025-0589en)
Supplement: Translation

# Translation: Orientações para terapia de reposição enzimática domiciliar em crianças e adolescentes com mucopolissacaridose: revisão de escopo^*^

**DOI:** 10.1590/1980-220X-REEUSP-2025-0589pt

**Authors:** Vinicius Rodrigues de Oliveira, Bárbara Letícia de Queiroz Xavier, Carlos Vinícius de Souza Felipe, Isabele Gouveia Muniz de Alencar, Hallyson Leno Lucas da Silva, Francisca Marta de Lima Costa Souza, Jonas Sâmi Albuquerque de Oliveira

**Affiliations:** 1 Universidade Federal do Rio Grande do Norte, Programa de Pós-Graduação em Enfermagem, Natal, RN, Brazil.; 2 Universidade Federal do Rio Grande do Norte, Programa de Pós-Graduação em Saúde Coletiva, Natal, RN, Brazil.; 3 Universidade Federal do Rio Grande do Norte, Curso de Medicina, Natal, RN, Brazil.; 4 Universidade Federal do Rio Grande do Norte, Departamento de Enfermagem, Natal, RN, Brazil.

**Keywords:** Mucopolissacaridoses, Terapia de Reposição de Enzimas, Criança, Adolescente, Enfermagem Domiciliar

## Abstract

**Objetivo:**

Identificar e mapear as principais orientações para realização da terapia de reposição enzimática (TRE) domiciliar administrada em crianças e adolescentes diagnosticados com mucopolissacaridose.

**Método:**

Revisão de escopo, conduzida segundo o *JBI Manual for Evidence Synthesis* (*Edition* 2024). Foram consultadas 12 fontes de dados com estratégias de busca específicas. Os dados foram extraídos por formulário próprio e analisados por estatística descritiva simples e abordagem qualitativa PAGER.

**Resultados:**

A amostra final foi composta por 13 artigos publicados entre 2007 e 2020, majoritariamente conduzidos em países europeus, com predominância de autores vinculados ao *Royal Manchester Children’s Hospital*. Foram identificados cinco padrões para a consolidação de uma assistência qualificada e segura no contexto da TRE domiciliar administrada em crianças e adolescentes com mucopolissacaridoses dos tipos I, II, IV-A e VII. A enfermeira emerge como profissional essencial em todas as etapas e atividades apresentadas.

**Conclusão:**

Apesar dos avanços acerca das orientações para descentralização da TRE ainda persistem desafios para sua implementação, dentre os quais se destaca a necessidade de disseminação do conhecimento técnicocientífico.

## INTRODUÇÃO

A mucopolissacaridose (MPS) é um grupo de doenças genéticas que decorre da incapacidade do organismo de produzir, de forma adequada, as enzimas responsáveis por degradar os glicosaminoglicanos (GAGs). Estes, por sua vez, desempenham funções biológicas essenciais e, quando não são degradados pela atividade lisossomal, acumulam-se no corpo. Esse acúmulo provoca graves complicações em diversos sistemas corporais, podendo levar ao óbito ainda nos primeiros anos de vida. Atualmente, são conhecidos sete tipos de MPS, classificados e catalogados de acordo com a enzima deficiente^(1,2)^.

O tratamento da MPS vem evoluindo ao longo do tempo; contudo, ainda não há uma cura definitiva para a doença. As intervenções terapêuticas atuais visam retardar a evolução da doença, mitigar os sintomas e melhorar a qualidade de vida dos pacientes. Diante desse cenário, a escolha do tratamento adequado deve considerar aspectos como o tipo da MPS, a idade do paciente, o envolvimento neurológico e as condições clínicas associadas. Além disso, o tratamento de escolha deve ser iniciado precocemente, ainda na infância, fase da doença em que seja possível prevenir complicações orgânicas^(2,3)^.

Um desses tratamentos é a Terapia de Reposição Enzimática (TRE) que, embora não seja uma solução definitiva é, atualmente, a mais acessível. A TRE surge na década de 1990, como alternativa exitosa para tratar a doença de Gaucher, e é comercializada nos anos 2000, com propósito de tratar a MPS dos tipos I, II, IV-A, VI e VII. Em termos gerais, a TRE objetiva suprir a carência da enzima ausente no corpo do paciente com MPS, por meio de infusões intravenosas que ocorrem semanalmente ou quinzenalmente. A enzima administrada conecta-se aos receptores de manose-6-fosfato (M6P) que se localizam na superfície das células, onde são direcionadas para os lisossomos, que por sua vez viabilizam a degradação dos GAGs^(4,5)^.

A administração da TRE é, geralmente, realizada em hospitais ou em centros de infusão e possui duração média de três a cinco horas. Esse cenário pode constituir barreiras à adesão terapêutica, ocasionar ausências escolares do paciente e perdas de jornada de trabalho pelos familiares, além de impactar significativamente a organização familiar e, consequentemente, repercutir de modo negativo na qualidade de vida dos envolvidos^(6)^.

Considerando tais limitações, surge a possibilidade da realização da TRE em domicílio, prática já implementada em países norte-americanos e europeus. A administração domiciliar da TRE oferece diversos benefícios, entre eles maior adesão ao tratamento, conforto e bem-estar, além de favorecer a satisfação com a terapia e a autogestão da doença^(7,8)^. Ademais, o atendimento domiciliar é particularmente benéfico no contexto do atendimento pediátrico, visto que este ambiente se apresenta como um espaço menos traumático, mais propício ao bem-estar e ao desenvolvimento da criança^(9)^.

Apesar dessas evidências, a TRE domiciliar ainda não é uma realidade consolidada no Brasil. No entanto, autores^(10)^ publicaram um consenso de especialistas brasileiros que reconhece essa prática como viável e segura no contexto da MPS dos tipos I, II e VI. De igual modo, o Ministério da Saúde do Brasil estabelece, por meio de Protocolos Clínicos e Diretrizes Terapêuticas (PCDT), orientações relacionadas à TRE^(11,12)^. Contudo, tais documentos apresentam orientações limitadas no que se refere à realização da TRE em ambiente domiciliar.

Diante disso, objetivou-se identificar e mapear as principais orientações para realização da terapia de reposição enzimática domiciliar administrada em crianças e adolescentes diagnosticados com mucopolissacaridose.

## MÉTODO

### Desenho do Estudo

Trata-se de um estudo do tipo revisão de escopo, fundamentado no arcabouço metodológico proposto pelo *JBI Manual for Evidence Synthesis* (*Edition* 2024)^(13)^ e relatado conforme as orientações do *Preferred Reporting Items for Systematic Reviews and Meta-Analyses for Scoping Reviews* (PRISMA-ScR)^(14)^. O protocolo deste estudo foi previamente registrado na *Open Science Framework* (OSF) e está disponível para consulta através do registro https://osf.io/qusb6/.

Adotou-se a seguinte pergunta de pesquisa: quais as principais orientações para realização da terapia de reposição enzimática domiciliar administrada em crianças e adolescentes diagnosticados com MPS? Ela foi elaborada com auxílio da estratégia População, Conceito e Contexto (PCC), em que: População (P) – crianças e adolescentes diagnosticados com MPS; Conceito (C) – orientações para terapia de reposição enzimática; Contexto (C) – ambiente domiciliar.

### Critérios de Elegibilidade

Foram considerados elegíveis como evidência: estudos primários, secundários, documentos oficiais (guias, manuais, protocolos), dissertações, teses, livros e anais de conferências científicas publicados em qualquer ano ou idioma desde que respondam à pergunta de pesquisa. Foram excluídas as evidências que não apresentaram texto completo disponível na íntegra, assim como estudos de reflexão, visto que não se adequaram aos aspectos metodológicos estabelecidos para esta revisão.

### Fontes de Dados e Estratégia de Busca

A pesquisa foi realizada em doze fontes de dados com abrangência internacional. A captação de literatura convencional foi realizada a partir das seguintes fontes: *Cumulative Index to Nursing and Allied Health Literature* (CINAHL via EBSCO), *Cochrane Library*, *Embase*, Literatura Latino-Americana e do Caribe em Ciências da Saúde (LILACS), *Medical Literature Analysis and Retrievel System Online* (Medline via Pubmed), *Science Direct*, *Scopus* e *Web of Science*.

Quanto à busca na literatura cinzenta foi realizada por meio do Catálogo de teses e dissertações da Fundação Coordenação de Aperfeiçoamento de Pessoal de Nível Superior (CAPES), do buscador *Google* Acadêmico, do *National Institute for Health and Care Excellence* (NICE) e do *World Health Organization Institutional Repository for Information Sharing* (WHO IRIS).

Para formulação da estratégia de busca de alta sensibilidade de acordo com cada fonte de dados, foram seguidos os passos: I) consulta aos Descritores em Ciências da Saúde (DeCS), *Medical Subject Headings* (MeSH) e *Emtree*; II) Verificação de termos alternativos/descritores não controlados; III) Organização dos descritores e termos alternativos em uma tabela no programa *Microsoft Office Word*, versão 2016; IV) Elaboração da estratégia de busca, fazendo a associação dos termos aos operadores booleanos “AND”, “OR” ou “NOT”. A estratégia final conforme cada fonte de dados está apresentada no Quadro 1.

**Quadro 1:** Estratégias de busca empregadas nas fontes de dados utilizadas – Natal, RN, Brasil, 2025.

Literatura convencional		
Fonte de dados	Estratégia de busca
CINAHL	(child OR children OR adolescent OR teen OR teenager OR youth) AND (mucopolysaccharidoses OR mucopolysaccharidosis) AND (“Enzyme Replacement Therapies” OR “Replacement Therapies, Enzyme” OR “Replacement Therapy, Enzyme” OR “Therapies, Enzyme Replacement” OR “Therapy, Enzyme Replacement”) AND (“Therapy, Home Infusion” OR “Home Infusion Therapies” OR “Infusion Therapies, Home” OR “Therapies, Home Infusion” OR “Infusion Therapy, Home”) NOT (Neoplasm NOT “Glycogen Storage Disease Type II” NOT “Gaucher Disease” NOT “Fabry Disease”)
Cochrane Library	Child OR Children in Title Abstract Keyword OR Adolescence OR Adolescents OR ‘Adolescents, Female’ OR ‘Adolescent, Female’ OR ‘Adolescent, Male’ OR ‘Male Adolescents’ OR Youth OR Youths OR Teen OR Teens OR Teenager OR Teenagers in Title Abstract Keyword AND mucopolysaccharidoses OR mucopolysaccharidosis in Title Abstract Keyword AND ‘Enzyme Replacement Therapy’ OR ‘Enzyme Replacement Therapies’ OR ‘Replacement Therapies, Enzyme’ OR ‘Replacement Therapy, Enzyme’ OR ‘Therapies, Enzyme Replacement’ OR ‘Therapy, Enzyme Replacement’ in Title Abstract Keyword OR ‘Home Infusion Therapy’ OR ‘Therapy, Home Infusion’ OR ‘Home Infusion Therapies’ OR ‘Infusion Therapies, Home’ OR ‘Therapies, Home Infusion’ OR ‘Infusion Therapy, Home’ in Title Abstract Keyword NOT Neoplasm NOT ‘Glycogen Storage Disease Type II’ NOT ‘Gaucher Disease’ NOT ‘Fabry Disease’ in Title Abstract Keyword
Embase	(‘child’/exp OR child OR ‘children’/exp OR children OR ‘adolescent’/exp OR ‘adolescent’) AND ‘mucopolysaccharidosis’ AND (‘enzyme replacement’ OR ‘enzyme replacement therapy’) AND ‘home infusion therapy’ NOT ‘neoplasm’ NOT ‘glycogen storage disease type 2’ NOT ‘gaucher disease’ NOT ‘fabry disease’
LILACS	mh:((criança OR adolescente) AND (mucopolissacaridoses) AND (“Terapia de Reposição de Enzimas”) OR (“Terapia por infusões no domicílio”) AND NOT (neoplasia) AND NOT (“Doença de Depósito de Glicogênio Tipo II”) AND NOT (“Doença de gaucher”) AND NOT (“Doença de fabry”))
Medline	(“child”[MeSH Terms] OR “child”[All Fields] OR “children”[All Fields] OR “child s”[All Fields] OR “children s”[All Fields] OR “childrens”[All Fields] OR “childs”[All Fields] OR (“child”[MeSH Terms] OR “child”[All Fields] OR “children”[All Fields] OR “child s”[All Fields] OR “children s”[All Fields] OR “childrens”[All Fields] OR “childs”[All Fields]) OR (“adolescences”[All Fields] OR “adolescency”[All Fields] OR “adolescent”[MeSH Terms] OR “adolescent”[All Fields] OR “adolescence”[All Fields] OR “adolescents”[All Fields] OR “adolescent s”[All Fields]) OR “adolescent*”[All Fields] OR “teen*”[All Fields] OR “teenager*”[All Fields] OR “youth*”[All Fields] OR “adolescent* female”[All Fields] OR “adolescent* male”[All Fields]) AND (“mucopolysaccharidose”[All Fields] OR “mucopolysaccharidoses”[MeSH Terms] OR “mucopolysaccharidoses”[All Fields] OR “mucopolysaccharidosis”[All Fields])) OR (“mucopolysaccharidose”[All Fields] OR “mucopolysaccharidoses”[MeSH Terms] OR “mucopolysaccharidoses”[All Fields] OR “mucopolysaccharidosis”[All Fields])) AND “Enzyme Replacement Therapy”[All Fields]) OR “Enzyme Replacement Therapies”[All Fields] OR “replacement therapies enzyme”[All Fields] OR “replacement therapy enzyme”[All Fields] OR “therapies enzyme replacement”[All Fields] OR “therapy enzyme replacement”[All Fields]) AND “Home Infusion Therapy”[All Fields]) OR (“Home Infusion Therapy”[MeSH Terms] OR (“home”[All Fields] AND “infusion”[All Fields] AND “therapy”[All Fields]) OR “Home Infusion Therapy”[All Fields] OR (“therapy”[All Fields] AND “home”[All Fields] AND “infusion”[All Fields])) OR “Home Infusion Therapies”[All Fields] OR (“Home Infusion Therapy”[MeSH Terms] OR (“home”[All Fields] AND “infusion”[All Fields] AND “therapy”[All Fields]) OR “Home Infusion Therapy”[All Fields] OR (“infusion”[All Fields] AND “therapies”[All Fields] AND “home”[All Fields])) OR (“Home Infusion Therapy”[MeSH Terms] OR (“home”[All Fields] AND “infusion”[All Fields] AND “therapy”[All Fields]) OR “Home Infusion Therapy”[All Fields] OR (“therapies”[All Fields] AND “home”[All Fields] AND “infusion”[All Fields])) OR “infusion therapy home”[All Fields]) NOT (“neoplasm”[All Fields]) NOT “Glycogen Storage Disease Type II”[All Fields]) NOT “Gaucher Disease”[All Fields]) NOT “Fabry Disease”[All Fields]
Science Direct	(Child AND Mucopolysaccharidoses AND “Enzyme Replacement Therapy” AND “Home Infusion Therapy” NOT Neoplasm NOT “Glycogen Storage Disease Type II” NOT “Gaucher Disease” NOT “Fabry Disease”)
Scopus	(TITLE-ABS-KEY (child OR children OR adolescent OR teen OR teenager OR youth) AND TITLE-ABS-KEY (mucopolysaccharidoses OR mucopolysaccharidosis) AND TITLE-ABS-KEY (“Enzyme Replacement Therapies” OR “Replacement Therapies, Enzyme” OR “Replacement Therapy, Enzyme” OR “Therapies, Enzyme Replacement” OR “Therapy, Enzyme Replacement”) AND TITLE-ABS-KEY (“Therapy, Home Infusion” OR “Home Infusion Therapies” OR “Infusion Therapies, Home” OR “Therapies, Home Infusion” OR “Infusion Therapy, Home”) AND NOT TITLE-ABS-KEY (neoplasm AND NOT “glycogen storage disease type iii” AND NOT “Gaucher Disease” AND NOT “Fabry Disease”))
Web of Science	child OR children OR adolescent OR Teen OR Teenager OR Youth (All Fields) AND Mucopolysaccharidoses OR Mucopolysaccharidosis (All Fields) AND ‘Enzyme Replacement Therapies’ OR ‘Replacement Therapies, Enzyme’ OR ‘Replacement Therapy, Enzyme’ OR ‘Therapies, Enzyme Replacement’ OR ‘Therapy, Enzyme Replacement’ (All Fields) AND ‘Therapy, Home Infusion’ OR ‘Home Infusion Therapies’ OR ‘Infusion Therapies, Home’ OR ‘Therapies, Home Infusion’ OR ‘Infusion Therapy, Home’ (All Fields) NOT Neoplasms NOT ‘Glycogen Storage Disease Type II’ NOT ‘Gaucher Disease’ NOT ‘Fabry Disease’ (All Fields)
Literatura cinzenta	
Fonte de dados	Estratégia de busca
Catálogo de teses e dissertações da CAPES	(Criança OR Adolescente) AND Mucopolissacaridoses AND (Terapia de Reposição de Enzimas OR Terapia de Reposição Enzimática) AND Terapia por Infusões no Domicílio.
*Google* Acadêmico	Child OR Adolescent AND Mucopolysaccharidoses AND “Enzyme Replacement Therapy” AND “Home Infusion Therapy”
NICE	Child OR Adolescent AND Mucopolysaccharidoses AND “Enzyme Replacement Therapy” AND “Home Infusion Therapy”
WHO IRIS	Child OR Adolescent AND Mucopolysaccharidoses AND “Enzyme Replacement Therapy” AND “Home Infusion Therapy”

Fonte: Elaborado pelos autores.

Apesar de o operador “NOT” não ser de uso convencional, pelo risco de limitar a amostra da revisão, sua inclusão mostrouse necessária, uma vez que a TRE pode ser desenvolvida em outros contextos de adoecimento. Assim, durante os testes para a construção da estratégia de busca, percebeu-se que, sem o uso desse operador, surgiam inúmeros estudos que não abordavam a TRE em crianças e adolescentes com MPS, gerando uma sobrecarga de resultados que não se adequavam ao escopo da pesquisa.

A busca de dados ocorreu em 21 fevereiro de 2025, por meio do Portal de Periódicos da CAPES acessando a Comunidade Acadêmica Federada (CAFe) e aconteceu de forma pareada por dois pesquisadores. É importante destacar que, antes de iniciar a fase de busca e coleta de dados, todos os membros da pesquisa foram submetidos a um treinamento prévio, ministrado pelo pesquisador principal, com objetivo de garantir a padronização dos procedimentos e a obtenção de resultados mais fidedignos por parte da equipe.

### Extração de Dados

As publicações encontradas nas fontes de dados foram exportadas para o *software Rayyan* ^(15)^ (versão 2024, *Computing Research Institute*, Doha, Catar), onde foi realizada a triagem inicial considerando os critérios de elegibilidade previamente estabelecidos, junto a leitura de títulos e resumos. A seleção foi realizada pelos mesmos dois pesquisadores que participaram da etapa anterior e para que não houvesse influência na seleção do material, ocorreu de forma cega entre si, ou seja, os revisores não tiveram acesso aos pareceres um do outro, até a finalização desta etapa.

Quanto aos conflitos na decisão de inclusão ou não de determinado estudo na amostra, a princípio buscou-se a resolução por meio de discussão entre os avaliadores. Nos casos em que a discordância persistiu, a decisão foi definida a partir da emissão do parecer final de um terceiro avaliador vinculado à equipe de pesquisa, sendo este enfermeiro, com doutorado na área de Enfermagem e experiência no cuidado a crianças e adolescentes com MPS.

Após a avaliação de elegibilidade dos estudos encontrados nas fontes de dados e resolução de seus conflitos, foi empreendida a busca manual de referências. Essa etapa consistiu na análise das listas bibliográficas dos artigos selecionados como parte da amostra final, para identificar referências adicionais que pudessem contribuir para o aprofundamento do estudo.

A extração de dados dos estudos selecionados para amostra foi realizada de forma independente, por meio da plataforma *Google Forms*, utilizando um formulário personalizado, elaborado especificamente para atender aos objetivos desta revisão.

O formulário foi organizado em duas seções. A primeira tratou da extração de dados referentes ao perfil bibliométrico dos estudos da amostra, em que foram coletadas as informações referentes aos autores, instituição de vínculo, periódico, ano de publicação dos estudos, país de realização da pesquisa, objetivo, tipo de documento e tipo de estudo. A segunda seção abordou o conteúdo referente às orientações para TRE domiciliar administrada em crianças e adolescente com MPS.

### Análise e Tratamento de Dados

Os dados obtidos foram analisados de forma quantitativa por meio da estatística descritiva simples (frequências relativa e absoluta) para descrever o perfil bibliométrico dos estudos incluídos na amostra e de forma qualitativa com o emprego da estrutura PAGER. Essa estratégia trata de uma abordagem sistemática desenvolvida para melhorar a análise e o relato de revisões de escopo.

A estrutura PAGER é um mnemônico que representa cinco domínios analíticos inter-relacionados: Padrões, Avanços, Lacunas, Evidências para a Prática e Recomendações de Pesquisa. Essa abordagem permite mapear de forma clara e acessível as principais descobertas temáticas, identificar progressos na área estudada, apontar deficiências no corpo de literatura existente e destacar implicações práticas e futuras direções de pesquisa^(16)^.

## RESULTADOS

### Apresentação da Seleção dos Estudos

Foram identificados 5132 registros, dos quais 5055 originaram-se das estratégias de busca nas fontes de dados, resultando em nove estudos incluídos. Outros 77 registros foram identificados por métodos adicionais, dos quais quatro foram selecionados. A amostra final foi composta por 13 artigos e a seleção detalhada dos estudos está apresentada na Figura 1.

**Figura 1:** Fluxograma de seleção dos estudos conforme recomendações PRISMA (2020). Natal, RN, Brasil, 2025.

### Perfil Bibliométrico e Detalhamento dos Estudos Que Compuseram A Amostra

Os artigos revisados foram publicados entre 2007 e 2020, mas com hiatos nos anos de 2012, 2014, 2016 e 2019. Observou-se maior concentração de publicações nos anos de 2008, 2010 e 2018, cada um representando 15,4% da amostra (n = 2), totalizando 46,2% dos estudos incluídos. Nos demais anos em que houve publicações, a frequência foi de aproximadamente 7,7% (n = 1).

Quanto à distribuição geográfica, observa-se que a maioria dos estudos é proveniente de países europeus, os quais representam, em conjunto, aproximadamente 69,3% (n = 9) da amostra, com destaque para o Reino Unido e a Itália. Em seguida, destaca-se a participação de países do continente americano, como Brasil, EUA e Canadá.

Quanto às bases de dados utilizadas para a recuperação dos estudos, destacaram-se *Scopus*, *Medline* e *Google* Acadêmico, perfazendo 69% (n = 9) da amostra, os demais estudos estavam publicados na plataforma Science Direct, no site oficial do Ministério da Saúde e na *Scientific Electronic Library Online* (SciELO). Ressalta-se que, embora a SciELO não tenha sido utilizada como fonte de dados na presente revisão, um dos artigos incluídos na amostra, identificado por meio da busca manual nas referências, encontrava-se disponível nessa base.

No que se refere aos periódicos em que os estudos foram publicados, evidenciou-se que a revista *Molecular Genetics and Metabolism* reuniu o maior número de publicações, correspondendo a aproximadamente 23,1% (n = 3) da amostra. Esse periódico é classificado como de alto impacto, segundo as métricas referentes ao ano de 2024; na base *Scopus,* possui percentil de 65% e apresenta fator de impacto 3.5, conforme análise do *Journal Citation Reports* (JCR). Os demais periódicos apresentaram participação individual de 7,7% (n = 1).

No tocante às afiliações institucionais dos autores, foi possível agrupar as instituições por núcleos, a saber: hospitais e outras instituições de saúde, universidades e órgãos de saúde ou conselho/sociedade de especialistas. Assim, identificouse que os autores que mais publicaram na área da TRE domiciliar estavam vinculados a hospitais (50%), em destaque o *Royal Manchester Children’s Hospital* com maior número de profissionais vinculados aos artigos da amostra.

A descrição acerca do conteúdo dos estudos selecionados para a amostra final da revisão está apresentada no Quadro 2.

**Quadro 2:** Descrição dos estudos selecionados para amostra - Natal, RN, Brasil, 2025.

Autor	Tipo de estudo	Tamanho da amostra	Objetivo	Resultados
Wikman-Jorgensen et al.^(17)^	Revisão sistemática com metanálise	42 estudos	Avaliar a eficácia da TRE na síndrome de Hunter em termos do impacto em variáveis clínicas e de segurança.	Os dados sugerem um efeito claro e consistente da TRE na síndrome de Hunter.
Brasil^(11)^	Protocolo clínico e Diretriz Terapêutica	N/l^*^	Revisar práticas diagnósticas e terapêuticas e incorporar as recomendações referentes ao tratamento medicamentoso com a TRE com laronidase.	Apresenta o conceito geral da MPS do tipo I e estabelece critérios de diagnóstico, critérios de inclusão e de exclusão, tratamento e mecanismos de regulação, controle e avaliação.
Brasil^(12)^	Protocolo clínico e Diretriz Terapêutica	N/l^*^	Revisar práticas diagnósticas e terapêuticas e incorporar as recomendações referentes ao tratamento medicamentoso com a TRE com idursulfase alfa.	Apresenta o conceito geral da MPS do tipo II e estabelece critérios de diagnóstico, critérios de inclusão e de exclusão, tratamento e mecanismos de regulação, controle e avaliação.
Finnigan et al.^(18)^	Relato de experiência	14 participantes	Revisar a experiência de um ambiente pediátrico do Reino Unido com o tratamento de TRE domiciliar em crianças com MPS tipo IV-A e demonstrar os critérios necessários para garantir um programa de tratamento domiciliar bem-sucedido.	Dois pacientes tiveram que retornar ao hospital devido a dificuldades com acesso venoso em casa, sendo que um retornou para casa após a substituição por um cateter totalmente implantado e o outro retornou às infusões em casa com o acesso venoso. Um outro paciente desenvolveu reação adversa relacionada à infusão, então retornou ao ambiente hospitalar para duas infusões enquanto seu regime de pré-medicação e incrementos de taxa foram alterados. Este paciente não teve mais reações dentro do ambiente de atendimento domiciliar.
Sestito et al.^(19)^	Revisão de literatura	N/l^*^	Realizar uma revisão de literatura sobre o tratamento com idursulfase em pacientes com síndrome de Hunter.	A eficácia e a segurança da terapia com idursulfase foram avaliadas e confirmadas em muitos relatos clínicos. O acompanhamento em longo prazo de pacientes em TRE demonstrou a importância do início precoce do tratamento com idursulfase. Quanto à terapia domiciliar, tem sido associada a um aumento na adesão do paciente em comparação ao tratamento hospitalar e a uma melhora na qualidade de vida tanto dos pacientes quanto de seus familiares.
Ceravolo et al.^(20)^	Relato de experiência	3 participantes	Relatar os resultados de uma experiência no sul da Itália com 3 pacientes com síndrome de Hunter que estão em tratamento domiciliar por 73 a 87 semanas.	Durante o primeiro ano de tratamento domiciliar, mais infusões foram administradas em casa do que no centro especializado. As pontuações do questionário sobre saúde e bem-estar (EQ-5D) dos pais dos pacientes aumentaram quando o paciente estava em tratamento domiciliar, indicando uma melhora importante na qualidade de vida.
Scarpa et al.^(21)^	Consenso de especialistas e revisão bibliográfica	26 especialistas	Descrever as recomendações desenvolvidas pelo Conselho Europeu de Especialistas em Síndrome de Hunter para o diagnóstico e tratamento da MPS tipo II.	As recomendações enfatizam a necessidade de abordagem multidisciplinar. A TRE com idursulfase ajuda a melhorar sinais somáticos, porém sem efeito sobre os sintomas neurológicos. A terapia em domicílio promove maior adesão do paciente, além de uma melhor qualidade de vida. Contudo, a transição para casa deve ser avaliada por protocolos rigorosos.
Burton et al.^(22)^	Relato de experiência	11 participantes	Relatar a experiência com 11 pacientes com distúrbios de MPS em TRE que foram transferidos om sucesso para infusões domiciliares por períodos que variam de 21 a 86 semanas.	A TRE domiciliar é segura para pacientes com MPS. Uma diferença estatisticamente significativa foi observada no número de infusões perdidas no centro de infusão versus o ambiente domiciliar. Todos os pacientes expressaram satisfação com a terapia em casa e nenhum deles apresentou reação adversa durante a TRE domiciliar. Oito dos pacientes que frequentam a escola retornaram a um horário de escola em tempo integral.
Giugliani et al.^(10)^	Consenso de especialistas	47 especialistas	Reunir e harmonizar as informações disponíveis sobre o tratamento das MPSs tipos I,II e VI.	Apresenta orientações acerca da TRE para o tratamento da MPS no Brasil.
Burton et al.^(23)^	Relato de experiência	92 participantes	Utilizar a experiência com pacientes no banco de dados observacional HOS - Hunter Outcome Survey para avaliar a viabilidade de infusões domiciliares de idursulfase para pacientes com MPS tipo II.	Os pacientes iniciaram a TRE domiciliar após uma mediana de 9 meses de idursulfase. A maioria tinha entre 5 e 11 anos na transferência. Seis pacientes interromperam a terapia domiciliar; quatro posteriormente retomaram as infusões domiciliares. Cinco reações relacionadas à infusão ocorreram em 2 dos 59 pacientes que receberam terapia domiciliar por pelo menos 12 meses. As reações foram classificadas como leves a moderadas. Todas as reações que ocorreram em casa foram prontamente tratadas em casa.
Bagewadi et al.^(24)^	Relato de experiência	23 participantes	Revisar a experiência dos autores com o tratamento de TRE domiciliar em MPSs tipos I,II e VI tipos II e IV.	Tanto o Elaprase quanto o Naglazyme podem ser administrados com segurança na casa do paciente, aliviando assim a carga do tratamento. Ao decidir a adequação do tratamento domiciliar, um protocolo robusto com seleção cuidadosa do paciente, bom acesso vascular e um plano de tratamento detalhado para reações adversas e anafilaxia são essenciais para o sucesso do tratamento em domicílio.
Cox-Brinkman et al.^(25)^	Estudo de coorte	17 participantes	Investigar a viabilidade e a segurança da terapia domiciliar para pacientes com MPS I na Holanda.	A TRE pode ser administrada de forma satisfatória e segura em casa tanto em crianças quanto em adultos com MPS I. Mais de 1000 infusões domiciliares não resultaram em nenhuma reação relacionada à infusão ou dificuldades técnicas prolongadas. Apenas dois pacientes apresentaram reação associada à infusão durante o período inicial de TRE hospitalar. Nenhum dos pacientes apresentou reação associada à infusão após 3 meses de TRE.
Wraith et al.^(26)^	Revisão clínica	N/l^*^	Fornecer uma visão geral das manifestações clínicas, diagnóstico e tratamento sintomático de pacientes com MPS tipo II e fornecer recomendações para o uso de TRE.	A TRE com idursulfase, demonstrou reduzir significativamente os níveis de GAGs urinários e melhorar sintomas clínicos da MPS, especialmente quando iniciada precocemente. Embora a TRE não atravesse a barreira hematoencefálica, ela representa um avanço importante no manejo da doença somática. O tratamento deve ser multidisciplinar, iniciado o mais cedo possível e adaptado às necessidades clínicas do paciente, incluindo opções domiciliares para casos estáveis.

Legenda: ^*^Não identificado no estudo.

Fonte: Elaborado pelo autor.

### Principais Orientações Para Terapia de Reposição Enzimática Domiciliar Administrada em Crianças e Adolescentes Diagnosticados com Mucopolissacaridose

A análise da amostra permitiu identificar cinco padrões fundamentais para o desenvolvimento de uma assistência qualificada e segura frente a TRE domiciliar administrada em criança e do adolescente com MPS. A partir de cada um dos padrões estabelecidos, foram observados avanços, lacunas, evidências e, sobretudo, as principais orientações para administração da TRE domiciliar. O Quadro 3 sintetiza os principais achados organizados de acordo com a estratégia PAGER.

**Quadro 3:** Estrutura *PAGER* aplicada ao contexto do estudo - Natal, RN, Brasil, 2025.

Padrões	Avanços	Lacunas	Evidências para prática	Recomendações
Avaliação prévia da criança ou do adolescente para tomar a decisão de iniciar a TRE domiciliar, com base em indicações e contraindicações^(10,12,17,18,17,18,19,20,21,22,23,24,25,26)^	Desenvolvimento de protocolos que auxiliam na tomada de decisão em transferir criança ou adolescente para TRE domiciliar.	Falta de padronização da literatura com relação a idade mínima e período prévio realizando a TRE em hospital/ambulatório antes de iniciá-la em domicílio.	A TRE domiciliar pode ser iniciada se a criança ou adolescente atende todos os seguintes critérios: - Idade igual ou maior a cinco anos (preferencialmente). - Iniciou a TRE há pelo menos seis meses em ambiente ambulatorial/hospitalar, sem apresentar reações adversas relacionadas à infusão. - Apresenta acesso venoso periférico de fácil punção ou possui cateter totalmente implantado. - Possui ambiente domiciliar que é favorável a realização da TRE. - Não possui doença respiratória significativa.	Realização de estudos com maior nível de evidência científica que permitam definir a idade ideal para transicionar a criança para a TRE domiciliar.
Orientações à família e o paciente antes de iniciar a TRE domiciliar^(18,20,22,23,24)^.	Orientações realizadas por profissionais de diferentes áreas e especialidades.	A maior parte dos estudos apresenta pouco detalhamento acerca das orientações que devem ser prestadas à família da criança ou adolescente que irá iniciar a TRE domiciliar.	Os familiares devem ser orientados quanto: - Possibilidade de realização das infusões no domicílio iniciarem somente quando os pais se sintam seguros e manifestem aceitação quanto à realização do tratamento em casa. - Necessidade de um ambiente adequado para realização de todos os processos que envolvem a infusão. - Benefícios da TRE domiciliar. - Riscos de reações relacionadas à infusão e das medidas para o gerenciamento dessas reações. - Possíveis riscos associados ao cateter totalmente implantado. - Fatores para interrupção do tratamento domiciliar.	Ampliar a perspectiva sociofamiliar em estudos que tratam da TRE domiciliar, apresentando uma abordagem mais ampla acerca do papel do cuidador e da importância das orientações aos familiares.
Preparo e administração da medicação para TRE domiciliar ^(10,11,12,18,24)^.	Não foram identificados avanços em relação ao preparo e à administração da TRE em domicílio. O processo, em geral, segue os mesmos protocolos estabelecidos para a realização da TRE em ambiente ambulatorial.	- Não há uma descrição ampla dos aspectos necessários para que o ambiente domiciliar seja considerado ideal para preparo de administração da TRE. - Os estudos não mencionam preparo e administração da medicação de modo personalizado para o ambiente domiciliar. Segue-se o padrão, conforme a administração ambulatorial ou hospitalar.	Aprovação, pela equipe médica e de enfermagem, do local para preparo e armazenamento da medicação. - Antes de iniciar a infusão, o profissional que acompanhará o procedimento deve: a) Realizar e registrar anamnese e exame físico b) Fornecer a pré-medicação, para prevenir reações adversas à infusão, conforme prescrição médica. - Durante o processo de preparo e administração devem-se utilizar técnicas assépticas adequadas. - Em relação ao preparo deve-se: a) Selecionar a quantidade de frascos da medicação considerando o peso do paciente e a dose padrão recomendada. b) Colocar os frascos em local externo a refrigeração até que alcancem a temperatura ambiente de forma natural, sem aquecê-los. c) Verificar o aspecto da medicação. Caso esteja alterado, orienta-se a não realizar a infusão. d) Estabelecer o volume total que o paciente receberá durante a infusão. e) Desprezar uma quantidade de soro fisiológico da bolsa equivalente ao volume que será retirado dos frascos de enzima, garantindo que, após a adição da enzima, o volume total da solução continue sendo de 100 mL ou 250 mL. f) Retirar vagarosamente dos frascos a quantidade adequada da enzima. É importante que a solução não seja agitada, pois a agitação pode desnaturar o produto e comprometer sua atividade biológica. g) Adicionar a enzima à bolsa com soro fisiológico de forma gradual. A bolsa com a solução pronta deve ser suavemente agitada para garantir que o medicamento se distribua de maneira uniforme. - A administração da medicação deve ocorrer imediatamente após a sua preparação. Caso isso não seja possível, a solução não deve permanecer em temperatura ambiente. Recomenda-se o armazenamento em geladeira, entre 2 °C e 8 °C. - A infusão deve ser administrada por via endovenosa, utilizando bomba de infusão. É necessário o uso de equipo apropriado para o dispositivo, associado a filtro de 0,2 pm.	Elaboração e validação de manuais ou protocolos para guiar administração da TRE, considerando as diferenças entre o ambiente domiciliar e hospitalar
Assistência de enfermagem à TRE domiciliar^(10,18,20,21,22,23,24,25)^.	Capacitações para melhorar a qualidade do atendimento.	- Falta de detalhamento acerca do conteúdo do treinamento das enfermeiras. - Não é especificado o número de pacientes que uma enfermeira de tratamento domiciliar pode gerenciar.	As enfermeiras são responsáveis por: - Observar uma seção de infusão enquanto o paciente ainda está sendo tratado no hospital. - Realizar visita prévia à casa do paciente e verificar a adequação do ambiente para realização da TRE e armazenamento dos medicamentos. - Promover reunião com os familiares, orientá-los e sanar suas dúvidas. - Preparar e administrar a medicação da TRE. - Acompanhar toda a seção de infusão no domicílio. - Gerenciar as reações adversas. - Encaminhar relatório clínico mensal para acompanhar a evolução clínica da doença dos pacientes. - Estar sempre em contato com o centro de infusão, principalmente em casos de reações adversas. - Comunicar ao médico da Atenção Primária à Saúde acerca do andamento das infusões e reações adversas.	Estabelecer normativas mais claras sobre a formação e os aspectos laborais relacionados à atuação da enfermeira que presta assistência a crianças e adolescentes em TRE domiciliar.
Gerenciamento das reações adversas^(10,12,18,19,22,23,24,26)^.	Rede de gerenciamento das reações adversas.	Não há especificação de intervalos de tempo em que cada medida deve ser tomada, ou em que momento o paciente deve ser reavaliado para implementação da ação seguinte.	A enfermeira deve estar adequadamente equipada para o manejo de possíveis reações adversas. - Em casos de reações leves, como calafrios, prurido, náusea, cefaleia e/ou irritabilidade (especialmente em crianças menores), o profissional responsável pela infusão deve: a) Diminuir a velocidade da infusão em 50%. b) Ofertar antipirético e anti-histamínico por via oral. c) Se o paciente ainda apresentar sintomas, diminuir em mais 50% a velocidade da infusão. d) Se persistirem os sintomas interromper definitivamente a infusão. - Em casos de reações moderadas, como febre, cefaleia grave, vômitos, dor torácica, dor abdominal e/ou urticária, o profissional responsável pela infusão deve: a) Interromper imediatamente a infusão. b) Administrar anti-histamínicos e corticoides por via intravenosa. c) Administrar broncodilatador, se previamente prescrito pelo médico. - Em casos de reações graves, como dispneia, edema de língua ou glote, choque anafilático ou cardiogênico, o profissional que acompanha a infusão deve: a) Interromper imediatamente a infusão. b) Acionar os serviços de emergência. c) Administrar epinefrina via intramuscular, além de anti-histamínicos e corticoides via intravenosa. d) Estabilizar e realizar a transferência para o hospital. e) Coletar sangue para realização do exame de IgE. - Em casos de reações adversas moderadas ou graves a infusão seguinte deverá acontecer em ambiente ambulatorial/hospitalar.	Criação ou adaptação de instrumentos de monitoramento clínico para aplicação no cenário de reações adversas relacionadas à TRE.

Fonte: Elaborado pelo autor.

Destaca-se como um achado de extrema relevância no âmbito desta revisão a identificação de orientações para a terapia domiciliar apenas para pacientes diagnosticados com MPS tipo I(10,11,17,25); MPS tipo II(10,12,19–22,24,26); MPS tipo IV-A^(18,22,24)^; MPS tipo VI^(10,22)^. Assim, as orientações aqui apresentadas estão limitadas a esses tipos de MPS.

## DISCuSSÃO

Os resultados deste estudo permitiram a identificação e o mapeamento das principais orientações que fundamentam a descentralização da TRE, administrada em crianças e adolescentes, para o ambiente domiciliar. Essas evidências contribuem para a implementação segura da TRE domiciliar, ao evidenciar aspectos que podem embasar a prática e a tomada de decisão dos profissionais de saúde envolvidos nesse processo, com ênfase no papel do enfermeiro.

A transição para o cuidado domiciliar requer uma avaliação minuciosa de múltiplos aspectos, sendo que, dentre eles, destacam-se como primordiais o quadro clínico do paciente e o ambiente domiciliar^(27)^. No contexto da TRE, os fatores supracitados não deixam de ser essenciais; no entanto, adicionam-se a eles outros elementos obrigatórios que levam em consideração as peculiaridades da criança ou adolescente com MPS^(18)^. Em caso de pacientes pediátricos, deve-se, incialmente, identificar sua faixa etária, para definir se há recomendação de iniciar a transição para os cuidados domiciliares.

Conforme a experiência de autores^(22)^, é possível realizar a transição segura da TRE para o domicílio em pacientes menores de cinco anos, enquanto outros autores^(19,21,24)^ reconhecem que há dificuldade de evidências sólidas que respaldem a decisão de iniciar a TRE domiciliar em crianças menores de cinco anos, porém esses autores não descartam a possibilidade de que o tratamento ocorra em casa, desde que haja uma avaliação médica criteriosa para embasar a decisão.

O número de infusões previamente realizadas em ambiente hospitalar também deve ser considerado. Recomenda-se que a criança ou o adolescente tenha passado por, no mínimo, três^(19,21,22,24,26)^ a seis meses^(11,12,22,25)^ de tratamento em regime hospitalar, uma vez que é frequente a ocorrência de reações adversas durante os primeiros meses de administração.

Embora não tenham sido encontrados estudos que retratem a experiência brasileira no contexto da TRE domiciliar, os profissionais podem seguir a recomendação dos PCDTs^(11,12)^ para MPS, do Ministério da Saúde, que orientam a realização de, no mínimo, seis meses de terapia em ambiente hospitalar antes da descentralização do tratamento para o domicílio.

Outro fator que pode influenciar a decisão de administrar a TRE em domicílio é o paciente possuir um cateter totalmente implantado. Autores^(25)^ relatam que, apesar do interesse em realizar a terapia domiciliar, a dificuldade de acesso venoso periférico levou à não inclusão de adolescentes nesse modelo de tratamento.

O uso do cateter torna-se necessário, uma vez que a obtenção do acesso periférico é dificultada pelas alterações corporais decorrentes da MPS^(28)^. Considera-se também que o uso de cateter totalmente implantado, além de facilitar o manejo do paciente na execução da terapia domiciliar, pode aumentar a adesão ao tratamento, uma vez que proporciona experiências menos estressantes para a criança.

Para confirmar a viabilidade da descentralização do tratamento para o domicílio ainda é necessário verificar se não há sinais de risco ou fatores que possam comprometer o tratamento. Estudos contraindicam a TRE domiciliar para pacientes portadores de doença respiratória com grau significativo, pois, nessas condições, há maiores riscos de complicações e difícil manejo delas. A classificação de doença respiratória com grau significativo pode ser obtida por meio da avaliação espirométrica, quando o resultado do teste de capacidade vital forçada (CVF) é de 40% ou menos, ou quando se observam evidências de doença obstrutiva grave das vias aéreas^(19,21,24,26)^.

Cumprida a etapa de verificação do perfil, deve-se avaliar se a família tem interesse em aderir a essa modalidade terapêutica. Estudos têm mostrado que, quando oportunizada, a maioria das famílias opta pelo tratamento em casa, dispondo-se a colaborar em diversos aspectos, inclusive auxiliando a enfermeira durante a infusão^(24,25)^. Entretanto, existem alguns casos em que familiares de crianças e adolescentes enfermos optam por manter o tratamento no âmbito hospitalar, decisão muitas vezes atrelada ao sentimento de despreparo para lidar com as demandas assistenciais^(29)^.

Dessa forma, emerge como ponto central do processo de transição do cuidado, a necessidade de orientações claras aos familiares. Em primeiro lugar, é fundamental assegurar que o domicílio disponha de um ambiente adequado, razão pela qual recomenda-se que o médico ou a enfermeira realize uma visita domiciliar para avaliar se estão presentes todos os requisitos necessários para a infusão, a saber: local para armazenamento da medicação, espaço para realizar a infusão e questões relativas à segurança do profissional^(10,24)^. Em território brasileiro essas recomendações são ratificadas pela ANVISA^(30)^.

Os familiares também devem ser devidamente informados acerca dos benefícios da terapia domiciliar, tais como a redução de gastos, a diminuição de deslocamentos, a redução de perdas relacionadas às atividades laborais e escolares, promoção de maior conforto ao paciente e melhoria da qualidade de vida(17,20,22,23).

Outra orientação refere-se ao risco de reações adversas. Embora estas ocorram em um número reduzido de pacientes, há possibilidade de manifestação, especialmente em crianças menores. A família deve ser informada de que o tratamento domiciliar pode ser temporariamente interrompido diante de reações adversas moderadas ou graves^(21,24)^.

Após a preparação da criança, do adolescente e de sua família, iniciam-se as etapas relacionadas à administração da TRE. Com base nos estudos analisados na presente revisão, não foram identificadas diferenças substanciais entre a preparação e administração da TRE no hospital e no domicílio. As recomendações são inicialmente realizar a anamnese e o exame físico do paciente, seguidos da administração da medicação prévia à infusão. Posteriormente, procede-se ao preparo das enzimas, empregando técnicas assépticas, rigorosa higienização das mãos e observância do aspecto da medicação^(10)^.

Faz-se necessário destacar que, embora essas condutas sejam validadas entre especialistas, é preciso considerar aspectos relacionados ao ambiente domiciliar, que pode apresentar diversas nuances. Por isso, recomenda-se o desenvolvimento de instrumentos que orientem a prática adequada do preparo e da administração da enzima, considerando este ambiente.

Frente às recomendações aqui apresentadas, pode-se afirmar que a enfermeira desempenha importantes atribuições no processo da TRE. Na literatura internacional a enfermeira é apontada como uma figura essencial para o sucesso da TRE e sua aproximação com o paciente contribui para que este tenha uma melhor experiência durante o tratamento^(18,20,22,24,25)^. Além das ações já apresentadas, ainda são vistas como função da enfermeira o gerenciamento de possíveis reações adversas, o encaminhamento de relatórios clínicos mensais e o contato regular com o médico da APS.

Assim, observa-se que os estudos que compuseram esta revisão direcionam ao entendimento de que TRE domiciliar demanda o desenvolvimento da Prática Avançada de Enfermagem. As enfermeiras de prática avançada são profissionais que dispõem de um arcabouço de conhecimentos especializados e desenvolvem competências para a tomada de decisão clínica complexa^(31)^.

Sobre o gerenciamento das reações adversas, a enfermeira irá atuar conforme a gravidade da reação, sendo que essas podem ser leves, moderadas ou graves. Nos quadros leves, a diminuição da taxa de infusão associada ao uso de antitérmicos e antihistamínicos geralmente é suficiente. Para casos que demonstrem uma gravidade maior, orienta-se a interrupção da infusão com a combinação de medicamentos de diferentes classes^(24)^. Nos casos em que ocorrem reações graves, a enfermeira também é responsável por coletar amostra de sangue para exame de IgE, analisando a possibilidade de iniciar protocolos de tolerância ao medicamento, conduzidos no serviço hospitalar^(11,12)^.

Um ponto de destaque foi a criação da rede de gerenciamento de reações adversas, experiência reportada por autores^(20)^. A referida rede é composta por hospitais, médicos da APS, enfermeiras e familiares dos pacientes, todos integrados ao processo da TRE domiciliar.

Além dos aspectos clínicos, cabe à enfermeira conduzir as questões gerenciais, como a produção de relatório semanal da TRE, encaminhando-o ao centro de infusão, além da comunicação contínua com o médico da APS. Essa articulação reafirma as diretrizes da Política Nacional de Atenção Integral às Pessoas com Doenças Raras^(32)^. Diante desse cenário, é imprescindível apontar que as múltiplas atribuições da enfermeira e uma demanda de cuidado complexo podem levar a rotinas extenuantes. Isso reforça a necessidade de estabelecer normativas específicas para a atuação da enfermeira no contexto da TRE domiciliar.

Quanto às limitações do estudo, os autores reconhecem que a literatura acerca da TRE domiciliar administrada em crianças e adolescentes com MPS é escassa, característica comum em pesquisas que tratam de terapias para doenças raras. A delimitação da faixa etária pode ter reduzido ainda mais a identificação de estudos, principalmente aqueles que incluem populações de diferentes idades. Além disso, a possível restrição de acesso a artigos não disponibilizados gratuitamente, mesmo com a busca realizada por meio do Portal de Periódicos da CAPES, pode ter limitado o alcance da busca, resultando na não inclusão de evidências potencialmente relevantes.

Observou-se ainda a ausência de publicações mais recentes, isto é, no período de 2021 a 2024, o que demonstra uma lacuna na literatura e reforça a necessidade de novas pesquisas que produzam evidências científicas atualizadas sobre a TRE domiciliar associada ao contexto da infância e adolescência.

O estudo contribui para o avanço da enfermagem ao destacar o papel protagonista da enfermeira na administração da TRE, tanto no ambiente hospitalar quanto no domicílio. Também oferece subsídios para apoiar os profissionais no processo de descentralização da TRE para o cuidado domiciliar, ao passo que suscita o debate acerca dos desafios envolvidos na transição de crianças e adolescentes diante desse novo contexto de cuidado.

## CONCLUSÃO

A execução da TRE domiciliar já ocorre em diversos países do mundo e possui experiências positivas crescentes em países da América Latina, mas timidamente no Brasil. Ainda que AD tenha se consolidado no país ao longo dos anos, sua associação à TRE demanda um conhecimento mais abrangente sobre a MPS e seu manejo clínico, muitas vezes limitado a um pequeno grupo de profissionais especialistas.

Embora tenham sido identificados avanços na articulação da TRE em ambiente domiciliar, que vão desde a definição de parâmetros para elegibilidade dos pacientes até o gerenciamento e execução do cuidado no domicílio, ainda persistem lacunas significativas que necessitam ser superadas em âmbito nacional, como a não sistematização desse conhecimento para implementação na prática assistencial.

Por fim, vale ressaltar que a enfermeira foi identificada como figura-chave no processo da TRE domiciliar. Assim, diante da complexidade e da extensão das atribuições desempenhadas por essa profissional nesse contexto, evidencia-se a necessidade de fortalecimento da Prática Avançada de Enfermagem, de modo a sustentar a tomada de decisão clínica qualificada. Ademais, aponta-se a necessidade do desenvolvimento de protocolos e outras ferramentas de cuidado que orientem a prática da TRE em domicílio.

## Data Availability

Todo o conjunto de dados que dá suporte aos resultados deste estudo foi publicado no próprio artigo.

# Translation: Guías para la terapia de reemplazo enzimático domiciliaria en niños y adolescentes con mucopolisacaridosis: una revisión del alcance*

**DOI:** 10.1590/1980-220X-REEUSP-2025-0589es

**Authors:** Vinicius Rodrigues de Oliveira, Bárbara Letícia de Queiroz Xavier, Carlos Vinícius de Souza Felipe, Isabele Gouveia Muniz de Alencar, Hallyson Leno Lucas da Silva, Francisca Marta de Lima Costa Souza, Jonas Sâmi Albuquerque de Oliveira

**Affiliations:** 1 Universidade Federal do Rio Grande do Norte, Programa de Pós-Graduação em Enfermagem, Natal, RN, Brasil.; 2 Universidade Federal do Rio Grande do Norte, Programa de Pós-Graduação em Saúde Coletiva, Natal, RN, Brasil.; 3 Universidade Federal do Rio Grande do Norte, Curso de Medicina, Natal, RN, Brasil.; 4 Universidade Federal do Rio Grande do Norte, Departamento de Enfermagem, Natal, RN, Brasil.

**Keywords:** Mucopolisacaridosis, Terapia de Reemplazo Enzimático, Niño; Adolescente, Cuidados de Enfermería en el Hogar

## Abstract

**Objetivo:**

Identificar y trazar un mapa de las principales directrices para llevar a cabo la terapia de reemplazo enzimático (TRE) domiciliaria administrada a niños y adolescentes diagnosticados con mucopolisacaridosis.

**Método:**

Revisión del alcance, realizada de acuerdo con *JBI Manual for Evidence Synthesis* (*Edition* 2024). Se consultaron 12 fuentes de datos utilizando estrategias de búsqueda específicas. Los datos se extrajeron utilizando un formulario específico y se analizaron mediante estadísticas descriptivas simples y un enfoque cualitativo tipo PAGER.

**Resultados:**

La muestra final consistió en 13 artículos publicados entre 2007 y 2020, realizados en su mayoría en países europeos, con predominio de autores afiliados a *Royal Manchester Children’s Hospital*. Se identificaron cinco patrones para consolidar una atención cualificada y segura en el contexto de la TRE domiciliaria administrada a niños y adolescentes con mucopolisacaridosis tipos I, II, IV-A y VII. La enfermera se erige como una profesional esencial en todas las etapas y actividades que se presentan.

**Conclusión:**

A pesar de los avances logrados en cuanto a las directrices para la descentralización de la TRE, persisten los desafíos para su implementación, entre los que destaca la necesidad de difusión de conocimientos técnicos y científicos.

## INTRODUCCIÓN

La mucopolisacaridosis (MPS) es un grupo de enfermedades genéticas que se deriva de la incapacidad del organismo para producir, de manera adecuada, las enzimas responsables de degradar los glicosaminoglicanos (GAG). Estos, a su vez, desempeñan funciones biológicas esenciales y, cuando no son degradados por la actividad lisosomal, se acumulan en el cuerpo. Esta acumulación provoca graves complicaciones en diversos sistemas corporales, pudiendo llevar a la muerte incluso en los primeros años de vida. Actualmente, se conocen siete tipos de MPS, clasificados y catalogados de acuerdo con la enzima deficiente^(1,2)^.

El tratamiento de la MPS ha ido evolucionando con el tiempo; sin embargo, aún no existe una cura definitiva para la enfermedad. Las intervenciones terapéuticas actuales tienen como objetivo retrasar la evolución de la enfermedad, mitigar los síntomas y mejorar la calidad de vida de los pacientes. Ante este panorama, la elección del tratamiento adecuado debe considerar aspectos como el tipo de MPS, la edad del paciente, la afectación neurológica y las afecciones clínicas asociadas.

Además, el tratamiento de elección debe iniciarse de manera temprana, aún en la infancia, etapa de la enfermedad en la que es posible prevenir complicaciones orgánicas^(2,3)^.

Uno de estos tratamientos es la Terapia de Reemplazo Enzimático (TRE) que, aunque no es una solución definitiva, es actualmente la más accesible. La TRE surgió en la década de 1990 como una alternativa exitosa para tratar la enfermedad de Gaucher, y se comercializó en la década de 2000 con el propósito de tratar la MPS de los tipos I, II, IV-A, VI y VII. En términos generales, la TRE tiene como objetivo suplir la carencia de la enzima ausente en el organismo del paciente con MPS, mediante infusiones intravenosas que se administran semanalmente o quincenalmente. La enzima administrada se une a los receptores de manosa-6-fosfato (M6P) que se encuentran en la superficie de las células, desde donde se dirigen a los lisosomas, los cuales a su vez permiten la degradación de los GAG^(4,5)^.

La administración de la TRE se realiza, por lo general, en hospitales o en centros de infusión y tiene una duración promedio de tres a cinco horas. Este escenario puede constituir un obstáculo para la adherencia al tratamiento, provocar ausencias escolares del paciente y pérdidas de jornada laboral para los familiares, además de impactar significativamente en la organización familiar y, en consecuencia, repercutir de manera negativa en la calidad de vida de los involucrados^(6)^.

Teniendo en cuenta estas limitaciones, surge la posibilidad de realizar la TRE en el hogar, una práctica ya implementada en países norteamericanos y europeos. La administración domiciliaria de la TRE ofrece diversos beneficios, entre ellos una mayor adherencia al tratamiento, comodidad y bienestar, además de favorecer la satisfacción con la terapia y el autocontrol de la enfermedad^(7,8)^. Además, la atención domiciliaria es particularmente beneficiosa en el contexto de la atención pediátrica, ya que este entorno se presenta como un espacio menos traumático, más propicio para el bienestar y el desarrollo del niño^(9)^.

A pesar de estas evidencias, la TRE domiciliaria aún no es una realidad consolidada en Brasil. Sin embargo, algunos autores^(10)^ han publicado un consenso de especialistas brasileños que reconoce esta práctica como viable y segura en el contexto de la MPS de los tipos I, II y VI. Del mismo modo, el Ministerio de Salud de Brasil establece, a través de Protocolos Clínicos y Directrices Terapéuticas (PCDT), orientaciones relacionadas con la TRE^(11,12)^. No obstante, dichos documentos presentan orientaciones limitadas en lo que se refiere a la realización de la TRE en el ámbito domiciliario.

Ante esto, se propuso identificar y mapear las principales orientaciones para la realización de la terapia de reemplazo enzimático domiciliaria administrada en niños y adolescentes diagnosticados con mucopolisacaridosis.

## MÉTODO

### Diseño del Estudio

Se trata de un estudio de revisión de alcance, basado en el marco metodológico propuesto por el *JBI Manual for Evidence Synthesis* (*Edición* 2024)^(13)^ y redactado de acuerdo con las orientaciones de los *Preferred Reporting Items for Systematic Reviews and Meta-Analyses for Scoping Reviews* (PRISMA-ScR)^(14)^.

El protocolo de este estudio se registró previamente en el *Open Science Framework* (OSF) y está disponible para su consulta a través del registro https://osf.io/qusb6/.

Se adoptó la siguiente pregunta de investigación: ¿cuáles son las principales orientaciones para la realización de la terapia de reemplazo enzimático domiciliaria administrada a niños y adolescentes diagnosticados con MPS? Esta se elaboró con la ayuda de la estrategia Población, Concepto y Contexto (PCC), en la que: Población (P) – niños y adolescentes diagnosticados con MPS; Concepto (C) – directrices para la terapia de reemplazo enzimático; Contexto (C) – entorno domiciliario.

### Criterios de Elegibilidad

Se consideraron elegibles como evidencia: estudios primarios, secundarios, documentos oficiales (guías, manuales, protocolos), disertaciones, tesis, libros y actas de congresos científicos publicados en cualquier año o idioma, siempre que respondieran a la pregunta de investigación. Se excluyeron las evidencias que no presentaban el texto completo disponible en su totalidad, así como los estudios de reflexión, ya que no se ajustaban a los aspectos metodológicos establecidos para esta revisión.

### Fuentes de Datos y Estrategia de Búsqueda

La búsqueda se realizó en doce fuentes de datos de alcance internacional. La recopilación de literatura convencional se llevó a cabo a partir de las siguientes fuentes: *Cumulative Index to Nursing and Allied Health Literature* (CINAHL vía EBSCO), *Cochrane Library*, *Embase*, Literatura Latinoamericana y del Caribe en Ciencias de la Salud (LILACS), *Medical Literature Analysis and Retrieval System Online* (Medline vía PubMed), *Science Direct*, *Scopus* y *Web of Science*.

En cuanto a la búsqueda en la literatura gris, se llevó a cabo a través del Catálogo de tesis y disertaciones de la Fundación Coordinación para el Perfeccionamiento del Personal de Nivel Superior (CAPES), del buscador *Google* Académico, del *National Institute for Health and Care Excellence* (NICE) y del *World Health Organization Institutional Repository for Information Sharing* (WHO IRIS).

Para formular la estrategia de búsqueda de alta sensibilidad de acuerdo con cada fuente de datos, se siguieron los siguientes pasos: I) consulta de los Descriptores en Ciencias de la Salud (DeCS), Medical Subject Headings (MeSH) y Emtree; II) verificación de términos alternativos/descriptores no controlados; III) Organización de los descriptores y términos alternativos en una tabla en el programa *Microsoft Office Word*, versión 2016; IV) Elaboración de la estrategia de búsqueda, asociando los términos a los operadores booleanos «AND», «OR» o «NOT». La estrategia final según cada fuente de datos se presenta en el Cuadro 1.

**Cuadro 1:** Estrategias de búsqueda empleadas en las fuentes de datos utilizadas – Natal, RN, Brasil, 2025.

Literatura convencional		
Fuente de datos	Estrategia de búsqueda
CINAHL	(child OR children OR adolescent OR teen OR teenager OR youth) AND (mucopolysaccharidoses OR mucopolysaccharidosis) AND (“Enzyme Replacement Therapies” OR “Replacement Therapies, Enzyme” OR “Replacement Therapy, Enzyme” OR “Therapies, Enzyme Replacement” OR “Therapy, Enzyme Replacement”) AND (“Therapy, Home Infusion” OR “Home Infusion Therapies” OR “Infusion Therapies, Home” OR “Therapies, Home Infusion” OR “Infusion Therapy, Home”) NOT (Neoplasm NOT “Glycogen Storage Disease Type II” NOT “Gaucher Disease” NOT “Fabry Disease”)
Cochrane Library	Child OR Children in Title Abstract Keyword OR Adolescence OR Adolescents OR ‘Adolescents, Female’ OR ‘Adolescent, Female’ OR ‘Adolescent, Male’ OR ‘Male Adolescents’ OR Youth OR Youths OR Teen OR Teens OR Teenager OR Teenagers in Title Abstract Keyword AND mucopolysaccharidoses OR mucopolysaccharidosis in Title Abstract Keyword AND ‘Enzyme Replacement Therapy’ OR ‘Enzyme Replacement Therapies’ OR ‘Replacement Therapies, Enzyme’ OR ‘Replacement Therapy, Enzyme’ OR ‘Therapies, Enzyme Replacement’ OR ‘Therapy, Enzyme Replacement’ in Title Abstract Keyword OR ‘Home Infusion Therapy’ OR ‘Therapy, Home Infusion’ OR ‘Home Infusion Therapies’ OR ‘Infusion Therapies, Home’ OR ‘Therapies, Home Infusion’ OR ‘Infusion Therapy, Home’ in Title Abstract Keyword NOT Neoplasm NOT ‘Glycogen Storage Disease Type II’ NOT ‘Gaucher Disease’ NOT ‘Fabry Disease’ in Title Abstract Keyword
Embase	(‘child’/exp OR child OR ‘children’/exp OR children OR ‘adolescent’/exp OR ‘adolescent’) AND ‘mucopolysaccharidosis’ AND (‘enzyme replacement’ OR ‘enzyme replacement therapy’) AND ‘home infusion therapy’ NOT ‘neoplasm’ NOT ‘glycogen storage disease type 2’ NOT ‘gaucher disease’ NOT ‘fabry disease’
LILACS	mh:((criança OR adolescente) AND (mucopolissacaridoses) AND (“Terapia de Reposição de Enzimas”) OR (“Terapia por infusões no domicílio”) AND NOT (neoplasia) AND NOT (“Doença de Depósito de Glicogênio Tipo II”) AND NOT (“Doença de gaucher”) AND NOT (“Doença de fabry”))
Medline	(“child”[MeSH Terms] OR “child”[All Fields] OR “children”[All Fields] OR “child s”[All Fields] OR “children s”[All Fields] OR “childrens”[All Fields] OR “childs”[All Fields] OR (“child”[MeSH Terms] OR “child”[All Fields] OR “children”[All Fields] OR “child s”[All Fields] OR “children s”[All Fields] OR “childrens”[All Fields] OR “childs”[All Fields]) OR (“adolescences”[All Fields] OR “adolescency”[All Fields] OR “adolescent”[MeSH Terms] OR “adolescent”[All Fields] OR “adolescence”[All Fields] OR “adolescents”[All Fields] OR “adolescent s”[All Fields]) OR “adolescent*”[All Fields] OR “teen*”[All Fields] OR “teenager*”[All Fields] OR “youth*”[All Fields] OR “adolescent* female”[All Fields] OR “adolescent* male”[All Fields]) AND (“mucopolysaccharidose”[All Fields] OR “mucopolysaccharidoses”[MeSH Terms] OR “mucopolysaccharidoses”[All Fields] OR “mucopolysaccharidosis”[All Fields])) OR (“mucopolysaccharidose”[All Fields] OR “mucopolysaccharidoses”[MeSH Terms] OR “mucopolysaccharidoses”[All Fields] OR “mucopolysaccharidosis”[All Fields])) AND “Enzyme Replacement Therapy”[All Fields]) OR “Enzyme Replacement Therapies”[All Fields] OR “replacement therapies enzyme”[All Fields] OR “replacement therapy enzyme”[All Fields] OR “therapies enzyme replacement”[All Fields] OR “therapy enzyme replacement”[All Fields]) AND “Home Infusion Therapy”[All Fields]) OR (“Home Infusion Therapy”[MeSH Terms] OR (“home”[All Fields] AND “infusion”[All Fields] AND “therapy”[All Fields]) OR “Home Infusion Therapy”[All Fields] OR (“therapy”[All Fields] AND “home”[All Fields] AND “infusion”[All Fields])) OR “Home Infusion Therapies”[All Fields] OR (“Home Infusion Therapy”[MeSH Terms] OR (“home”[All Fields] AND “infusion”[All Fields] AND “therapy”[All Fields]) OR “Home Infusion Therapy”[All Fields] OR (“infusion”[All Fields] AND “therapies”[All Fields] AND “home”[All Fields])) OR (“Home Infusion Therapy”[MeSH Terms] OR (“home”[All Fields] AND “infusion”[All Fields] AND “therapy”[All Fields]) OR “Home Infusion Therapy”[All Fields] OR (“therapies”[All Fields] AND “home”[All Fields] AND “infusion”[All Fields])) OR “infusion therapy home”[All Fields]) NOT (“neoplasm”[All Fields]) NOT “Glycogen Storage Disease Type II”[All Fields]) NOT “Gaucher Disease”[All Fields]) NOT “Fabry Disease”[All Fields]
Science Direct	(Child AND Mucopolysaccharidoses AND “Enzyme Replacement Therapy” AND “Home Infusion Therapy” NOT Neoplasm NOT “Glycogen Storage Disease Type II” NOT “Gaucher Disease” NOT “Fabry Disease”)
Scopus	( TITLE-ABS-KEY ( child OR children OR adolescent OR teen OR teenager OR youth ) AND TITLE-ABS-KEY ( mucopolysaccharidoses OR mucopolysaccharidosis ) AND TITLE-ABS-KEY ( “Enzyme Replacement Therapies” OR “Replacement Therapies, Enzyme” OR “Replacement Therapy, Enzyme” OR “Therapies, Enzyme Replacement” OR “Therapy, Enzyme Replacement” ) AND TITLE-ABS-KEY ( “Therapy, Home Infusion” OR “Home Infusion Therapies” OR “Infusion Therapies, Home” OR “Therapies, Home Infusion” OR “Infusion Therapy, Home” ) AND NOT TITLE-ABS-KEY ( neoplasm AND NOT “glycogen storage disease type iii” AND NOT “Gaucher Disease” AND NOT “Fabry Disease” ) )
Web of Science	child OR children OR adolescent OR Teen OR Teenager OR Youth (All Fields) AND Mucopolysaccharidoses OR Mucopolysaccharidosis (All Fields) AND ‘Enzyme Replacement Therapies’ OR ‘Replacement Therapies, Enzyme’ OR ‘Replacement Therapy, Enzyme’ OR ‘Therapies, Enzyme Replacement’ OR ‘Therapy, Enzyme Replacement’ (All Fields) AND ‘Therapy, Home Infusion’ OR ‘Home Infusion Therapies’ OR ‘Infusion Therapies, Home’ OR ‘Therapies, Home Infusion’ OR ‘Infusion Therapy, Home’ (All Fields) NOT Neoplasms NOT ‘Glycogen Storage Disease Type II’ NOT ‘Gaucher Disease’ NOT ‘Fabry Disease’ (All Fields)
Literatura gris	
Fuente de datos	Estrategia de búsqueda
Catálogo de teses e dissertações da CAPES	(Criança OR Adolescente) AND Mucopolissacaridoses AND (Terapia de Reposição de Enzimas OR Terapia de Reposição Enzimática) AND Terapia por Infusões no Domicílio.
*Google* Acadêmico	Child OR Adolescent AND Mucopolysaccharidoses AND “Enzyme Replacement Therapy” AND “Home Infusion Therapy”
NICE	Child OR Adolescent AND Mucopolysaccharidoses AND “Enzyme Replacement Therapy” AND “Home Infusion Therapy”
WHO IRIS	Child OR Adolescent AND Mucopolysaccharidoses AND “Enzyme Replacement Therapy” AND “Home Infusion Therapy”

Fuente: Elaborado por los autores.

Aunque el operador «NOT» no es de uso convencional, debido al riesgo de limitar la muestra de la revisión, su inclusión resultó necesaria, ya que la TRE puede desarrollarse en otros contextos de enfermedad. Así, durante las pruebas para la construcción de la estrategia de búsqueda, se observó que, sin el uso de este operador, surgían numerosos estudios que no abordaban la TRE en niños y adolescentes con MPS, generando una sobrecarga de resultados que no se ajustaban al alcance de la investigación.

La búsqueda de datos se llevó a cabo el 21 de febrero de 2025 a través del Portal de Revistas de CAPES, accediendo a la Comunidad Académica Federada (CAFe), y fue realizada de forma emparejada por dos investigadores. Es importante destacar que, antes de iniciar la fase de búsqueda y recolección de datos, todos los miembros del equipo de investigación recibieron una capacitación previa, impartida por el investigador principal, con el objetivo de garantizar la estandarización de los procedimientos y la obtención de resultados más confiables por parte del equipo.

### Extracción de Datos

Las publicaciones encontradas en las fuentes de datos se exportaron al *software Rayyan* ^(15)^ (versión 2024, *Computing Research Institute*, Doha, Catar), donde se llevó a cabo la selección inicial teniendo en cuenta los criterios de elegibilidad previamente establecidos, junto con la lectura de títulos y resúmenes. La selección fue realizada por los mismos dos investigadores que participaron en la etapa anterior y, para que no hubiera influencia en la selección delmaterial, se llevó a cabo de forma ciega entre ellos, es decir, los revisores no tuvieron acceso a las opiniones del otro hasta la finalización de esta etapa.

En cuanto a los conflictos en la decisión de incluir o no un determinado estudio en la muestra, en principio se buscó la resolución mediante la discusión entre los evaluadores. En los casos en que persistió el desacuerdo, la decisión se definió a partir del dictamen final de un tercer evaluador vinculado al equipo de investigación, el cual era un enfermero con doctorado en el área de Enfermería y experiencia en el cuidado de niños y adolescentes con MPS.

Tras la evaluación de la elegibilidad de los estudios encontrados en las fuentes de datos y la resolución de sus conflictos, se llevó a cabo la búsqueda manual de referencias. Esta etapa consistió en el análisis de las listas bibliográficas de los artículos seleccionados como parte de la muestra final, para identificar referencias adicionales que pudieran contribuir a la profundización del estudio.

La extracción de datos de los estudios seleccionados para la muestra se realizó de forma independiente, a través de la plataforma *Google Forms*, utilizando un formulario personalizado, elaborado específicamente para cumplir con los objetivos de esta revisión.

El formulario se organizó en dos secciones. La primera se ocupó de la extracción de datos relativos al perfil bibliométrico de los estudios de la muestra, en la que se recopiló la información relativa a los autores, la institución de afiliación, la revista, el año de publicación de los estudios, el país donde se realizó la investigación, el objetivo, el tipo de documento y el tipo de estudio. La segunda sección abordó el contenido referente a las orientaciones para la TRE domiciliaria administrada en niños y adolescentes con MPS.

### Análisis y Tratamiento de Datos

Los datos obtenidos se analizaron de forma cuantitativa mediante estadística descriptiva simple (frecuencias relativa y absoluta) para describir el perfil bibliométrico de los estudios incluidos en la muestra y de forma cualitativa con el empleo de la estructura PAGER. Esta estrategia consiste en un enfoque sistemático desarrollado para mejorar el análisis y la presentación de informes de revisiones de alcance.

La estructura PAGER es un mnemotécnico que representa cinco dominios analíticos interrelacionados: Patrones, Avances, Gaps, Evidencias para la Práctica y Recomendaciones de Investigación.

Este enfoque permite mapear de manera clara y accesible los principales hallazgos temáticos, identificar avances en el área estudiada, señalar deficiencias en el corpus de literatura existente y destacar implicaciones prácticas y futuras direcciones de investigación^(16)^.

## RESULTADOS

### Presentación de la Selección de Estudios

Se identificaron 5132 registros, de los cuales 5055 procedían de las estrategias de búsqueda en las fuentes de datos, lo que dio como resultado la inclusión de nueve estudios. Se identificaron otros 77 registros mediante métodos adicionales, de los cuales se seleccionaron cuatro. La muestra final estuvo compuesta por 13 artículos y la selección detallada de los estudios se presenta en la Figura 1.

**Figura 1:** Fluxograma de seleção dos estudos conforme recomendações PRISMA (2020). Natal, RN, Brasil, 2025.

### Perfil Bibliométrico y Detalles de los Estudios que Integraron la Muestra

Los artículos analizados se publicaron entre 2007 y 2020, aunque con interrupciones en los años 2012, 2014, 2016 y 2019. Se observó una mayor concentración de publicaciones en los años 2008, 2010 y 2018, cada uno de los cuales representó el 15,4% de la muestra (n = 2), lo que totaliza el 46,2% de los estudios incluidos. En los demás años en los que hubo publicaciones, la frecuencia fue de aproximadamente el 7,7% (n = 1).

En cuanto a la distribución geográfica, se observa que la mayoría de los estudios provienen de países europeos, los cuales representan, en conjunto, aproximadamente el 69,3% (n = 9) de la muestra, con destaque para el Reino Unido e Italia. A continuación, se destaca la participación de países del continente americano, como Brasil, EE. UU. y Canadá. En cuanto a las bases de datos utilizadas para la recuperación de los estudios, se destacaron *Scopus*, *Medline* y *Google*Académico, que representaron el 69% (n = 9) de la muestra; los demás estudios se publicaron en la plataforma Science Direct, en el sitio web oficial del Ministerio de Salud y en la *Scientific Electronic Library Online* (SciELO). Cabe destacar que, aunque SciELO no se utilizó como fuente de datos en la presente revisión, uno de los artículos incluidos en la muestra, identificado mediante una búsqueda manual en las referencias, se encontraba disponible en dicha base.

En cuanto a las revistas en las que se publicaron los estudios, se observó que la revista *Molecular Genetics and Metabolism* reunió el mayor número de publicaciones, lo que corresponde a aproximadamente el 23,1% (n = 3) de la muestra. Esta revista está clasificada como de alto impacto, según las métricas correspondientes al año 2024; en la base *Scopus,* tiene un percentil del 65% y presenta un factor de impacto de 3,5, según el análisis del *Journal Citation Reports* (JCR). Las demás revistas presentaron una participación individual del 7,7% (n = 1).

En cuanto a las afiliaciones institucionales de los autores, fue posible agrupar las instituciones por núcleos, a saber: hospitales y otras instituciones de salud, universidades y organismos de salud o consejos/sociedades de especialistas. Así, se identificó que los autores que más publicaron en el área de la TRE domiciliaria estaban vinculados a hospitales (50%), destacando el *Royal Manchester Children’s Hospital*, con el mayor número de profesionales vinculados a los artículos de la muestra.

La descripción del contenido de los estudios seleccionados para la muestra final de la revisión se presenta en el Cuadro 2.

**Cuadro 2:** Descripción de los estudios seleccionados para la muestra. Natal, RN, Brasil, 2025.

Autor	Tipo de estudio	Tamaño de la muestra	Objetivo	Resultados
Wikman-Jorgensen et al.^(7)^	Revisión sistemática con metaanálisis	42 estudios	Evaluar la eficacia de la TRE en el síndrome de Hunter en cuanto a su impacto en las variables clínicas y de seguridad.	Los datos sugieren un efecto claro y consistente de la TRE en el síndrome de Hunter.
Brasil^(11)^	Protocolo clínico y guía terapéutica	N/l*	Revisar las prácticas diagnósticas y terapéuticas e incorporar las recomendaciones relativas al tratamiento farmacológico con la TRE con laronidasa.	Presenta el concepto general de la MPS tipo I y establece criterios de diagnóstico, criterios de inclusión y exclusión, tratamiento y mecanismos de regulación, control y evaluación.
Brasil^(12)^	Protocolo clínico y guía terapéutica	N/l*	Revisar las prácticas diagnósticas y terapéuticas e incorporar las recomendaciones relativas al tratamiento farmacológico con la TRE con idursulfasa alfa.	Presenta el concepto general de la MPS tipo II y establece criterios de diagnóstico, criterios de inclusión y exclusión, tratamiento y mecanismos de regulación, control y evaluación.
Finnigan et al.^(18)^	Relato de experiencia	14 participantes	Revisar la experiencia de un entorno pediátrico del Reino Unido con el tratamiento de la TRE domiciliaria en niños con MPS tipo IV-A y demostrar los criterios necesarios para garantizar un programa de tratamiento domiciliario exitoso.	Dos pacientes tuvieron que regresar al hospital debido a dificultades con el acceso venoso en el hogar; uno regresó a casa tras la sustitución por un catéter totalmente implantado y el otro reanudó las infusiones en el hogar con el acceso venoso. Otro paciente desarrolló una reacción adversa relacionada con la infusión, por lo que regresó al entorno hospitalario para recibir dos infusiones mientras se modificaban su régimen de premedicación y los incrementos de la velocidad de infusión. Este paciente no volvió a presentar reacciones en el entorno de atención domiciliaria.
Sesti to et al.^(19)^	Revisión bibliográfica	N/l*	Realizar una revisión bibliográfica sobre el tratamiento con idursulfasa en pacientes con síndrome de Hunter.	La eficacia y la seguridad de la terapia con idursulfasa se han evaluado y confirmado en numerosos informes clínicos. El seguimiento a largo plazo de pacientes en TRE ha demostrado la importancia del inicio temprano del tratamiento con idursulfasa. En cuanto a la terapia domiciliaria, se ha asociado con un aumento en la adherencia del paciente en comparación con el tratamiento hospitalario y con una mejora en la calidad de vida tanto de los pacientes como desús familiares.
Ceravolo et al.^(20)^	Relato de experiencia	3 participantes	Informar sobre los resultados de una experiencia en el sur de Italia con 3 pacientes con síndrome de Hunter que están en tratamiento domiciliario desde hace entre 73 y 87 semanas.	Durante el primer año de tratamiento domiciliario, se administraron más infusiones en el hogar que en el centro especializado. Las puntuaciones del cuestionario sobre salud y bienestar (EQ-5D) de los padres de los pacientes aumentaron cuando el paciente recibía tratamiento domiciliario, lo que indica una mejora importante en la calidad de vida.
Scarpa et al.^(21)^	Consenso de expertos y revisión bibliográfica	26 especialistas	Describir las recomendaciones elaboradas por el Consejo Europeo de Especialistas en Síndrome de Hunter para el diagnóstico y tratamiento de la MPS tipo II.	Las recomendaciones enfatizan la necesidad de un enfoque multidisciplinario. La TRE con idursulfasa ayuda a mejorar los signos somáticos, pero no tiene efecto sobre los síntomas neurológicos. La terapia en el hogar promueve una mayor adherencia del paciente, además de una mejor calidad de vida. Sin embargo, la transición al hogar debe evaluarse mediante protocolos rigurosos.
Burton et al.^(22)^	Relato de experiencia	11 participantes	Informar sobre la experiencia con 11 pacientes con trastornos de MPS en TRE que fueron transferidos con éxito a infusiones domiciliarias durante períodos que varían de 21 a 86 semanas.	La TRE domiciliaria es segura para los pacientes con MPS. Se observó una diferencia estadísticamente significativa en el número de infusiones perdidas en el centro de infusión frente al entorno domiciliario. Todos los pacientes expresaron satisfacción con la terapia en casa y ninguno de ellos presentó reacciones adversas durante la TRE domiciliaria. Ocho de los pacientes que asisten a la escuela regresaron a un horario escolar de tiempo completo.
Giugliani et al.^(10)^	Consenso de expertos	47 participantes	Recopilar y armonizar la información disponible sobre el tratamiento de las MPS tipos I, II Y VI.	Presenta orientaciones sobre la TRE para el tratamiento de la MPS en Brasil.
Burton et al.(23)	Relato de experiencia	92 participantes	Utilizar la experiencia con pacientes en la base de datos observacional HOS (Hunter Outcome Survey) para evaluar la viabilidad de las infusiones domiciliarias de idursulfasa en pacientes con MPS tipo II.	Los pacientes iniciaron la TRE domiciliaria tras una mediana de 9 meses de idursulfasa. La mayoría tenía entre 5 y 11 años en el momento del cambio. Seis pacientes interrumpieron la terapia domiciliaria; cuatro reanudaron posteriormente las infusiones domiciliarias. Se produjeron cinco reacciones relacionadas con la infusión en 2 de los 59 pacientes que recibieron terapia domiciliaria durante al menos 12 meses. Las reacciones se clasificaron como leves a moderadas. Todas las reacciones que se produjeron en casa se trataron rápidamente en el hogar.
Bagewadi et al.^(24)^	Relato de experiencia	23 participantes	Revisar la experiencia de los autores con el tratamiento de la TRE domiciliaria en las mucopolisacaridosis tipos II y IV.	Tanto Elaprase como Naglazyme pueden administrarse de forma segura en el domicilio del paciente, aliviando así la carga del tratamiento. A la hora de decidir la idoneidad del tratamiento domiciliario, un protocolo sólido con una selección cuidadosa del paciente, un buen acceso vascular y un plan de tratamiento detallado para las reacciones adversas y la anafilaxia son esenciales para el éxito del tratamiento en el hogar.
Cox-Brinkman et al.^(25)^	Estudio de cohorte	17 participantes	Investigar la viabilidad y la seguridad de la terapia domiciliaria para pacientes con MPS tipo I en los Países Bajos.	La TRE puede administrarse de manera satisfactoria y segura en el hogar tanto en niños como en adultos con MPS tipo I. Más de 1000 infusiones domiciliarias no dieron lugar a ninguna reacción relacionada con la infusión ni a dificultades técnicas prolongadas. Solo dos pacientes presentaron una reacción asociada a la infusión durante el período inicial de TRE hospitalaria. Ninguno de los pacientes presentó una reacción asociada a la infusión después de 3 meses de TRE.
Wraith et al.^(26)^	Revisión clínica	N/l*	Ofrecer una visión general de las manifestaciones clínicas, el diagnóstico y el tratamiento sintomático de los pacientes con MPS tipo II, y proporcionar recomendaciones sobre el uso de la TRE.	La TRE con idursulfasa ha demostrado reducir significativamente los niveles de GAG urinarios y mejorar los síntomas clínicos de la MPS, especialmente cuando se inicia de manera temprana. Aunque la TRE no atraviesa la barrera hematoencefálica, representa un avance importante en el manejo de la enfermedad somática. El tratamiento debe ser multidisciplinario, iniciarse lo antes posible y adaptarse a las necesidades clínicas del paciente, incluyendo opciones domiciliarias para los casos estables.

Nota: ^*^No identificado en el studio.

Fuente: Elaborado por el autor.

### Principales Orientaciones para la Terapia de Reemplazo Enzimático Domiciliaria Administrada A Niños y Adolescentes Diagnosticados con Mucopolisacaridosis

El análisis de la muestra permitió identificar cinco patrones fundamentales para el desarrollo de una atención calificada y segura en el marco de la TRE domiciliaria administrada a niños y adolescentes con MPS. A partir de cada uno de los patrones establecidos, se observaron avances, gaps, evidencia y, sobre todo, las principales orientaciones para la administración de la TRE domiciliaria. El Cuadro 3 sintetiza los principales hallazgos organizados de acuerdo con la estrategia PAGER.

**Cuadro 3:** Estructura *PAGER* aplicada al contexto del studio – Natal, RN, Brasil, 2025.

Patrones	Avances	Gaps	Evidencias para práctica	Recomendaciones
Evaluación previa del niño o adolescente para decidir si se inicia la TRE domiciliaria, basándose en las indicaciones y contraindicacione^(10,12,17,18,19,20,21,22,23,24,25,26)^.	Desarrollo de protocolos que facilitan la toma de decisiones a la hora de derivar a un niño 0 adolescente a una TRE domiciliaria.	Falta de estandarización en la literatura con respecto a la edad mínima y el período previo de realización de la TRE en el hospital o en el consultorio antes de iniciarla en el hogar.	La TRE domiciliaria puede iniciarse si el niño o adolescente cumple todos los criterios siguientes: - Edad igual o superior a cinco años (preferiblemente). - Ha iniciado la TRE hace al menos seis meses en un entorno ambulatorio u hospitalario, sin presentar reacciones adversas relacionadas con la infusión. - Presenta un acceso venoso periférico de fácil punción o cuenta con un catéter totalmente implantado. - Dispone de un entorno domiciliario favorable para la realización de la TRE. - No padece ninguna enfermedad respiratoria significativa.	Realización de estudios con un mayor nivel de evidencia científica que permitan definir la edad ideal para que el niño pase a la TRE en el hogar.
Orientaciones para la familia y el paciente antes de iniciar la TRE domiciliaria^(18,20,22,24)^.	Orientaciones impartidas por profesionales de diferentes áreas y especialidades.	La mayoría de los estudios ofrecen pocos detalles sobre las orientaciones que se deben brindar a la familia del niño o adolescente que va a iniciar la TRE en el hogar.	Se debe orientar a los familiares sobre: - La posibilidad de iniciar las infusiones en el hogar solo cuando los padres se sientan seguros y expresen su aceptación respecto a la realización del tratamiento en casa. - La necesidad de un entorno adecuado para llevar a cabo todos los procesos relacionados con la infusión. - Los beneficios de la TRE domiciliaria. - Los riesgos de reacciones relacionadas con la infusión y las medidas para el manejo de dichas reacciones. - Los posibles riesgos asociados al catéter totalmente implantado. - Los factores que pueden llevar a la interrupción del tratamiento domiciliario.	Ampliar la perspectiva sociofamiliar en los estudios que tratan sobre la TRE en el hogar, presentando un enfoque más amplio sobre el papel del cuidador y la importancia de la orientación a los familiares.
Preparación y administración de la medicación para el tratamiento de la ETR en el hogar^(10,12,18,19,20,21,22,23,24)^.	No se identificaron avances en cuanto a la preparación y la administración de la TRE en el domicilio. El proceso, en general, sigue los mismos protocolos establecidos para la realización de la TRE en el ámbito ambulatorio.	No existe una descripción exhaustiva de los aspectos necesarios para que el entorno doméstico se considere ideal para la preparación de la administración de la TRE.	Aprobación, por parte del equipo médico y de enfermería, del lugar destinado a la preparación y almacenamiento de la medicación. - Antes de iniciar la infusión, el profesional que supervisará el procedimiento debe: a) Realizar y registrar la anamnesis y el examen físico b) Administrar la premedicación, para prevenir reacciones adversas a la infusión, según la prescripción médica. - Durante el proceso de preparación y administración se deben utilizar técnicas asépticas adecuadas. - En cuanto a la preparación, se debe: a) Seleccionar la cantidad de frascos de medicamento considerando el peso del paciente y la dosis estándar recomendada. b) Colocar los frascos en un lugar fuera del refrigerador hasta que alcancen la temperatura ambiente de forma natural, sin calentarlos. c) Verificar el aspecto del medicamento. En caso de que esté alterado, se recomienda no realizar la infusión. d) Establecer el volumen total que el paciente recibirá durante la infusión. e) Descartar de la bolsa una cantidad de Suero fisiológico equivalente al volumen que se extraerá de los frascos de enzima, asegurándose de que, tras la adición de la enzima, el volumen total de la solución siga siendo de TOO mL o 250 mL. f) Extraer lentamente de los frascos la cantidad adecuada de enzima. Es importante que la solución no se agite, ya que la agitación puede desnaturalizar el producto y comprometer su actividad biológica. g) Agregar la enzima a la bolsa con Suero fisiológico de manera gradual. La bolsa con la solución preparada debe agitarse suavemente para garantizar que el medicamento se distribuya de manera uniforme. - La administración del medicamento debe realizarse inmediatamente después de su preparación. Si esto no es posible, la solución no debe permanecer a temperatura ambiente. Se recomienda almacenarla en el refrigerador, entre 2 °C y 8 °C. - La infusión debe administrarse por vía intravenosa, utilizando una bomba de infusión. Es necesario el uso de equipo adecuado para el dispositivo, junto con un filtro de 0,2 μm	Elaboración y validación de manuales o protocolos para orientar la administración de la TRE, teniendo en cuenta las diferencias entre el entorno doméstico y el hospitalario.
Atención de enfermería para la TRE domiciliaria^(10,18,20,21,22,23,24,25)^.	Cursos de capacitación para mejorar la calidad del servicio.	- Falta de detalles sobre el contenido de la capacitación de las enfermeras. - No se especifica el número de pacientes que puede atender una enfermera de cuidados a domicilio.	Las enfermeras se encargan de: - Supervisar una sesión de infusión mientras el paciente aún se encuentra en tratamiento en el hospital. - Realizar una visita previa al domicilio del paciente y verificar que el entorno sea adecuado para llevar a cabo la TRE y almacenar los medicamentos. - Organizar una reunión con los familiares, orientarlos y aclarar sus dudas. - Preparar y administrar la medicación de la TRE. - Supervisar toda la sesión de infusión en el domicilio. - Manejar las reacciones adversas. - Enviar un informe clínico mensual para dar seguimiento a la evolución clínica de la enfermedad de los pacientes. - Estar siempre en contacto con el centro de infusión, especialmente en casos de reacciones adversas. - Informar al médico de atención primaria sobre el desarrollo de las infusiones y las reacciones adversas.	Establecer normas más claras sobre la formación y los aspectos laborales relacionados con la labor de las enfermeras que prestan asistencia a niños y adolescentes en el tratamiento de la epilepsia a domicilio.
Manejo de las reacciones adversas^(10,12,18,19,22,23,24,26)^.	Red de gestión de reacciones adversas	No se especifican los intervalos de tiempo en los que se debe tomar cada medida, ni en qué momento se debe reevaluar al paciente para poner en práctica la siguiente acción.	- La enfermera debe estar debidamente equipada para manejar posibles reacciones adversas. - En casos de reacciones leves, como escalofríos, prurito, náuseas, cefalea y/o irritabilidad (especialmente en niños pequeños), el profesional responsable de la infusión debe: a) Reducir la velocidad de la infusión en un 50%. b) Administrar un antipirético y un antihistamínico por vía oral. c) Si el paciente sigue presentando síntomas, reducir la velocidad de la infusión en un 50% más. c) Si los síntomas persisten, interrumpir definitivamente la infusión. - En casos de reacciones moderadas, como fiebre, cefalea grave, vómitos, dolor torácico, dolor abdominal y/o urticaria, el profesional responsable de la infusión debe: a) Interrumpir inmediatamente la infusión. b) Administrar antihistamínicos y corticoides por vía intravenosa. c) Administrar un broncodilatador, si ha sido prescrito previamente por el médico. - En casos de reacciones graves, como disnea, edema de la lengua o la glotis, shock anafiláctico o cardiogénico, el profesional que supervisa la infusión debe: a) Interrumpir inmediatamente la infusión. b) Llamar a los servicios de emergencia. c) Administrar epinefrina por vía intramuscular, además de antihistamínicos y corticoides por vía intravenosa. d) Estabilizar al paciente y trasladarlo al hospital. e) Realizar la recolección de sangre para realizar el examen de IgE. - En casos de reacciones adversas moderadas o graves, la siguiente infusión deberá realizarse en un entorno ambulatorio u hospitalario.	Creación o adaptación de instrumentos de seguimiento clínico para su aplicación en el contexto de las reacciones adversas relacionadas con la TRE.

Fuente: Elaborado por el autor.

Cabe destacar como un hallazgo de extrema relevancia en el ámbito de esta revisión la identificación de orientaciones para la terapia domiciliaria solo para pacientes diagnosticados con MPS tipo I^(10,11,17,25)^; MPS tipo II^(10,12,19,20,21,22,24,26)^; MPS tipo IV-A^(18,22,24)^; MPS tipo VI^(10,22)^. Por lo tanto, las orientaciones aquí presentadas se limitan a estos tipos de MPS.

## DISCUSIÓN

Los resultados de este estudio permitieron identificar y mapear las principales orientaciones que fundamentan la descentralización de la TRE, administrada en niños y adolescentes, hacia el entorno domiciliario. Estas evidencias contribuyen a la implementación segura de la TRE domiciliaria, al poner de manifiesto aspectos que pueden fundamentar la práctica y la toma de decisiones de los profesionales de la salud involucrados en este proceso, con énfasis en el papel del enfermero.

La transición a la atención domiciliaria requiere una evaluación minuciosa de múltiples aspectos, entre los que destacan como fundamentales el cuadro clínico del paciente y el entorno domiciliario^(27)^. En el contexto de la TRE, los factores mencionados anteriormente no dejan de ser esenciales; sin embargo, a ellos se suman otros elementos obligatorios que toman en cuenta las particularidades del niño o adolescente con MPS^(18)^. En el caso de pacientes pediátricos, se debe, en primer lugar, identificar su grupo de edad, para definir si existe la recomendación de iniciar la transición a la atención domiciliaria.

Según la experiencia de los autores^(22)^, es posible realizar una transición segura de la TRE al hogar en pacientes menores de cinco años, mientras que otros autores^(19,21,24)^ reconocen que es difícil encontrar evidencia sólida que respalde la decisión de iniciar la TRE domiciliaria en niños menores de cinco años; sin embargo, estos autores no descartan la posibilidad de que el tratamiento se realice en casa, siempre y cuando exista una evaluación médica minuciosa que sustente la decisión.

También debe tenerse en cuenta el número de infusiones realizadas previamente en el entorno hospitalario. Se recomienda que el niño o adolescente haya pasado por un mínimo de tres^(19,21,22,24,26)^ a seis meses^(11,12,22,25)^ de tratamiento en régimen hospitalario, ya que es frecuente la aparición de reacciones adversas durante los primeros meses de administración.

Aunque no se han encontrado estudios que reflejen la experiencia brasileña en el contexto de la TRE domiciliaria, los profesionales pueden seguir la recomendación de los PCDT^(11,12)^ para la MPS, del Ministerio de Salud, que orientan a realizar, como mínimo, seis meses de terapia en entorno hospitalario antes de la descentralización del tratamiento al domicilio.

Otro factor que puede influir en la decisión de administrar la TRE en el hogar es que el paciente cuente con un catéter totalmente implantado. Los autores^(25)^ informan que, a pesar del interés en realizar la terapia domiciliaria, la dificultad de acceso venoso periférico llevó a no incluir a adolescentes en este modelo de tratamiento.

El uso del catéter se vuelve necesario, ya que la obtención del acceso periférico se ve dificultada por las alteraciones corporales derivadas de la MPS^(28)^. También se considera que el uso de un catéter totalmente implantado, además de facilitar el manejo del paciente en la ejecución de la terapia domiciliaria, puede aumentar la adherencia al tratamiento, ya que proporciona experiencias menos estresantes para el niño.

Para confirmar la viabilidad de la descentralización del tratamiento al hogar, aún es necesario verificar que no haya signos de riesgo o factores que puedan comprometer el tratamiento. Los estudios contraindican la TRE domiciliaria para pacientes con enfermedad respiratoria de grado significativo, ya que, en estas condiciones, existe un mayor riesgo de complicaciones y un manejo difícil de las mismas. La clasificación de enfermedad respiratoria de grado significativo puede obtenerse mediante la evaluación espirométrica, cuando el resultado de la prueba de capacidad vital forzada (CVF) es del 40% o menos, o cuando se observan indicios de enfermedad obstructiva grave de las vías respiratorias(19,21,24,26).

Una vez completada la etapa de verificación del perfil, se debe evaluar si la familia tiene interés en adherirse a esta modalidad terapéutica. Los estudios han demostrado que, cuando se les ofrece la oportunidad, la mayoría de las familias opta por el tratamiento en el hogar, mostrándose dispuestas a colaborar en diversos aspectos, incluso ayudando a la enfermera durante la infusión^(24,25)^. Sin embargo, existen algunos casos en los que los familiares de niños y adolescentes enfermos optan por mantener el tratamiento en el ámbito hospitalario, decisión a menudo ligada al sentimiento de falta de preparación para lidiar con las exigencias asistenciales^(29)^.

De este modo, surge como punto central del proceso de transición de la atención la necesidad de orientaciones claras para los familiares. En primer lugar, es fundamental garantizar que el domicilio cuente con un entorno adecuado, por lo que se recomienda que el médico o la enfermera realicen una visita domiciliaria para evaluar si se cumplen todos los requisitos necesarios para la infusión, a saber: lugar para el almacenamiento de la medicación, espacio para realizar la infusión y cuestiones relacionadas con la seguridad del profesional^(10,24)^. En territorio brasileño, estas recomendaciones están ratificadas por la ANVISA^(30)^.

Los familiares también deben ser debidamente informados sobre los beneficios de la terapia domiciliaria, tales como la reducción de gastos, la disminución de los desplazamientos, la reducción de las pérdidas relacionadas con las actividades laborales y escolares, la promoción de un mayor confort para el paciente y la mejora de la calidad de vida^(17,20,22,23)^.

Otra orientación se refiere al riesgo de reacciones adversas. Aunque estas se producen en un número reducido de pacientes, existe la posibilidad de que se manifiesten, especialmente en niños pequeños. Se debe informar a la familia de que el tratamiento domiciliario puede interrumpirse temporalmente ante reacciones adversas moderadas o graves^(21,24)^.

Tras la preparación del niño, del adolescente y de su familia, se inician las etapas relacionadas con la administración de la TRE. A partir de los estudios analizados en la presente revisión, no se identificaron diferencias sustanciales entre la preparación y la administración de la TRE en el hospital y en el hogar. Las recomendaciones son, en primer lugar, realizar la anamnesis y el examen físico del paciente, seguidos de la administración de la medicación previa a la infusión. Posteriormente, se procede a la preparación de las enzimas, empleando técnicas asépticas, una higiene rigurosa de las manos y la observación del aspecto de la medicación^(10)^.

Cabe destacar que, aunque estas conductas están validadas entre los especialistas, es necesario considerar aspectos relacionados con el entorno domiciliario, que puede presentar diversos matices. Por ello, se recomienda el desarrollo de instrumentos que orienten la práctica adecuada de la preparación y la administración de la enzima, teniendo en cuenta este entorno.

A la luz de las recomendaciones aquí presentadas, se puede afirmar que la enfermera desempeña funciones importantes en el proceso de la TRE. En la literatura internacional, se señala a la enfermera como una figura esencial para el éxito de la TRE y su acercamiento al paciente contribuye a que este tenga una mejor experiencia durante el tratamiento^(18,20,22,24,25)^. Además de las acciones ya presentadas, también se consideran funciones de la enfermera el manejo de posibles reacciones adversas, el envío de informes clínicos mensuales y el contacto regular con el médico de APS.

Así, se observa que los estudios que componen esta revisión apuntan a la comprensión de que la TRE domiciliaria exige el desarrollo de la Práctica Avanzada de Enfermería. Las enfermeras de práctica avanzada son profesionales que cuentan con un marco de conocimientos especializados y desarrollan competencias para la toma de decisiones clínicas complejas^(31)^.

En cuanto al manejo de las reacciones adversas, la enfermera actuará según la gravedad de la reacción, que puede ser leve, moderada o grave. En los casos leves, la disminución de la velocidad de infusión junto con el uso de antipiréticos y antihistamínicos suele ser suficiente. Para los casos que presenten una gravedad mayor, se recomienda interrumpir la infusión y combinar medicamentos de diferentes clases^(24)^. En los casos en que se producen reacciones graves, la enfermera también es responsable de tomar una muestra de sangre para el examen de IgE, analizando la posibilidad de iniciar protocolos de tolerancia al medicamento, llevados a cabo en el servicio hospitalario^(11,12)^.

Un punto destacado fue la creación de la red de manejo de reacciones adversas, experiencia descrita por los autores^(20)^. Dicha red está compuesta por hospitales, médicos de APS, enfermeras y familiares de los pacientes, todos integrados en el proceso de la TRE domiciliaria.

Además de los aspectos clínicos, corresponde a la enfermera gestionar los asuntos administrativos, como la elaboración del informe semanal de la TRE, enviándolo al centro de infusión, además de la comunicación continua con el médico de APS. Esta articulación reafirma las directrices de la Política Nacional de Atención Integral a las Personas con Enfermedades Raras^(32)^. Ante este escenario, es imprescindible señalar que las múltiples atribuciones de la enfermera y una demanda de cuidados complejos pueden dar lugar a rutinas extenuantes. Esto refuerza la necesidad de establecer normativas específicas para la actuación de la enfermera en el contexto de la TRE domiciliaria.

En cuanto a las limitaciones del estudio, los autores reconocen que la literatura sobre la TRE domiciliaria administrada a niños y adolescentes con MPS es escasa, una característica común en las investigaciones que tratan sobre terapias para enfermedades raras. La delimitación del rango de edad puede haber reducido aún más la identificación de estudios, principalmente aquellos que incluyen poblaciones de diferentes edades. Además, la posible restricción de acceso a artículos no disponibles gratuitamente, incluso con la búsqueda realizada a través del Portal de Revistas de CAPES, puede haber limitado el alcance de la búsqueda, lo que ha dado lugar a la no inclusión de evidencia potencialmente relevante.

También se observó la ausencia de publicaciones más recientes, es decir, del período 2021 a 2024, lo que demuestra una laguna en la literatura y refuerza la necesidad de nuevas investigaciones que produzcan evidencia científica actualizada sobre la TRE domiciliaria asociada al contexto de la infancia y la adolescencia.

El estudio contribuye al avance de la enfermería al destacar el papel protagonista de la enfermera en la administración de la TRE, tanto en el entorno hospitalario como en el hogar. También ofrece recursos para apoyar a los profesionales en el proceso de descentralización de la TRE hacia la atención domiciliaria, al tiempo que suscita el debate sobre los desafíos que implica la transición de los niños y adolescentes ante este nuevo contexto de atención.

## CONCLUSIÓN

La implementación de la TRE domiciliaria ya se lleva a cabo en varios países del mundo y cuenta con experiencias positivas cada vez mayores en países de América Latina, aunque de manera tímida en Brasil. Si bien la atención domiciliaria se ha consolidado en el país a lo largo de los años, su asociación con la TRE exige un conocimiento más amplio sobre la MPS y su manejo clínico, que a menudo se limita a un pequeño grupo de profesionales especialistas.

Aunque se han identificado avances en la articulación de la TRE en el entorno domiciliario, que van desde la definición de parámetros de elegibilidad de los pacientes hasta la gestión y ejecución de la atención en el hogar, aún persisten gaps significativas que deben superarse a nivel nacional, como la falta de sistematización de este conocimiento para su implementación en la práctica asistencial.

Por último, cabe destacar que se identificó a la enfermera como una figura clave en el proceso de la TRE domiciliaria. Así, ante la complejidad y la amplitud de las funciones que desempeña esta profesional en este contexto, se pone de manifiesto la necesidad de fortalecer la Práctica Avanzada de Enfermería, a fin de respaldar la toma de decisiones clínicas calificadas. Además, se señala la necesidad de desarrollar protocolos y otras herramientas de atención que orienten la práctica de la TRE en el hogar.

## Data Availability

Todo el conjunto de datos que respalda los resultados de este estudio se publicó en el propio artículo.
